# Impact of a new balanced gelatine on electrolytes and pH in the perioperative care

**DOI:** 10.1371/journal.pone.0213057

**Published:** 2019-04-29

**Authors:** Gernot Marx, Patrick Meybohm, Tobias Schuerholz, Gösta Lotz, Mandy Ledinko, Achim W. Schindler, Rolf Rossaint, Kai Zacharowski

**Affiliations:** 1 Department of Intensive Care Medicine and Intermediate Care, University Hospital RWTH Aachen, Medical Faculty RWTH Aachen University, Aachen, Germany; 2 Department of Anesthesiology, Intensive Care Medfiicine & Pain Therapy, University Hospital Frankfurt, Frankfurt, Germany; 3 Department of Anaesthesiology and Intensive Care Medicine, University Hospital Rostock, Rostock, Germany; 4 Department of Anesthesiology, University Hospital RWTH Aachen, Medical Faculty RWTH University Aachen, Aachen, Germany; Public Library of Science, UNITED KINGDOM

## Abstract

**Introduction:**

Balanced fluid replacement solutions can possibly reduce the risks for electrolyte imbalances, for acid-base imbalances, and thus for renal failure. To assess the intraoperative change of base excess (BE) and chloride in serum after treatment with either a balanced gelatine/electrolyte solution or a non-balanced gelatine/electrolyte solution, a prospective, controlled, randomized, double-blind, dual centre phase III study was conducted in two tertiary care university hospitals in Germany.

**Material and methods:**

40 patients of both sexes, aged 18 to 90 years, who were scheduled to undergo elective abdominal surgery with assumed intraoperative volume requirement of at least 15 mL/kg body weight gelatine solution were included. Administration of study drug was performed intravenously according to patients need. The trigger for volume replacement was a central venous pressure (CVP) minus positive end-expiratory pressure (PEEP) <10 mmHg (CVP <10 mmHg). The crystalloid:colloid ratio was 1:1 intra- and postoperatively. The targets for volume replacement were a CVP between 10 and 14 mmHg minus PEEP after treatment with vasoactive agent and mean arterial pressure (MAP) > 65 mmHg.

**Results:**

The primary endpoints, intraoperative changes of base excess –2.59 ± 2.25 (median: –2.65) mmol/L (balanced group) and –4.79 ± 2.38 (median: –4.70) mmol/L (non-balanced group)) or serum chloride 2.4 ± 1.9 (median: 3.0) mmol/L and 5.2 ± 3.1 (median: 5.0) mmol/L were significantly different (p = 0.0117 and p = 0.0045, respectively). In both groups (each n = 20) the investigational product administration in terms of volume and infusion rate was comparable throughout the course of the study, i.e. before, during and after surgery.

**Discussion:**

Balanced gelatine solution 4% combined with a balanced electrolyte solution demonstrated significant smaller impact on blood gas analytic parameters in the primary endpoints BE and serum chloride when compared to a non-balanced gelatine solution 4% combined with NaCl 0.9%. No marked treatment differences were observed with respect to haemodynamics, coagulation and renal function.

**Trial registration:**

ClinicalTrials.gov (NCT01515397) and clinicaltrialsregister.eu, EudraCT number 2010-018524-58.

## Introduction

During surgery volume therapy is one of the most important features. The assessment of intravascular volume is extremely difficult and challenging. While for the usual perioperative situation crystalloids are the option of choice, acute hypovolaemia or shock are indications for the use of colloids in order to minimise the duration of hypoperfusion and consecutive tissue hypoxia. Many studies investigating the value of advanced haemodynamic monitoring in high risk surgical patients demonstrated that using algorithm based colloid resuscitation in combination with inotropic support is associated with a significant reduction of cardiac events and morbidity in general.[1–2] In a retrospective analysis of more than 100,000 patients Khuri et al. demonstrated the importance of a single life-threatening complication on long-term survival after surgery.[3]

The discussion on the use of hydroxyethylstarch (HES) in ICU patients induced a pharmaco-vigilance procedure of the European Medicines Agency, in which the concerns of the risks associated with the application of HES were also discussed for the perioperative care of patients resulting in a restriction for HES to be used only in the treatment of hypovolaemia due to acute blood loss, when crystalloids alone are not considered sufficient. Thus, gelatine is frequently used as an alternative colloid solution to treat hypovolaemia in the perioperative area. In abdominal surgery, for example, intra- and postoperative volume replacement is required. Haisch et al. showed that gelatine solutions are as effective as HES solutions used in abdominal surgery patients.[4]

Furthermore, the use of balanced solutions has been shown to be another important step forward for volume therapy. There is a good body of evidence available, that the use of balanced solutions instead of 0.9% saline results into less metabolic derangements, e.g. hyperchloraemic induced acidosis.[5] In consequence, the use of balanced volume solutions, crystalloids and/or colloids, are strongly recommended for the perioperative area in recent guidelines, such as NICE [6] or the German S3 guideline.[7]

Recently, a new balanced gelatine solution has been developed, but so far no clinical data on the use of that gelatine solution were available. Therefore, the primary objective of the study was to assess the intra-operative change of the BE and chloride after treatment of a balanced volume replacement regimen (balanced gelatine solution combined with a balanced electrolyte solution) compared with a non-balanced volume replacement regimen (non-balanced gelatine solution combined with a non-balanced electrolyte solution) in adult patients undergoing elective major abdominal surgery. The primary variable of the study was to show differences in the change of the BE and chloride (baseline–immediately after end of surgery) after treatment of the two volume replacement regimens.

Secondary objectives were safety and efficacy of the two different volume replacement regimens expressed by parameters such as arterial blood gas analysis, coagulation status, renal function, requirements of blood products, adverse events, haemodynamics, clinical outcome, demographic data, and surgery related data.

## Materials and methods

### Study design

This was a prospective, controlled, randomized, double-blind, bicentric phase III study performed in 2 parallel groups (EU Clinical Trials Register at https://www.clinicaltrialsregister.eu, EudraCT number 2010-018524-58 and at ClinicalTrials.gov (NCT01515397). It was sponsored and funded by B.Braun Melsungen AG, (Melsungen Germany). The registration was completed after the first inclusion for no specific reason; however, the initiation at about the turn of the year might have contributed to the delay. The authors confirm that all ongoing and related trials under their responsibility for this drug are registered. This study was performed in compliance with ICH Good Clinical Practice (CPMP/ICH/135/95). Comparison was made of an investigational test product containing 4% modified fluid gelatin in a balanced electrolyte solution (Gelaspan 4%, B. Braun Melsungen AG, Melsungen Germany) combined with a standard balanced electrolyte solution (Sterofundin ISO, B. Braun Melsungen AG, Melsungen Germany) with an investigational reference product containing 4% modified fluid gelatin in NaCl 0.9% (Gelafundin 4%, B. Braun Melsungen AG, Melsungen Germany) combined with a standard non-balanced electrolyte solution (NaCl 0.9%, B. Braun Melsungen AG, Melsungen Germany). Blinding was achieved by an independent pharmacy providing all solutions in undistinguishable flasks. The primary variable of the study was to show differences in the change of the BE and chloride (baseline–immediately after end of surgery) after treatment of the two volume replacement regimens.

**Fig 1:**
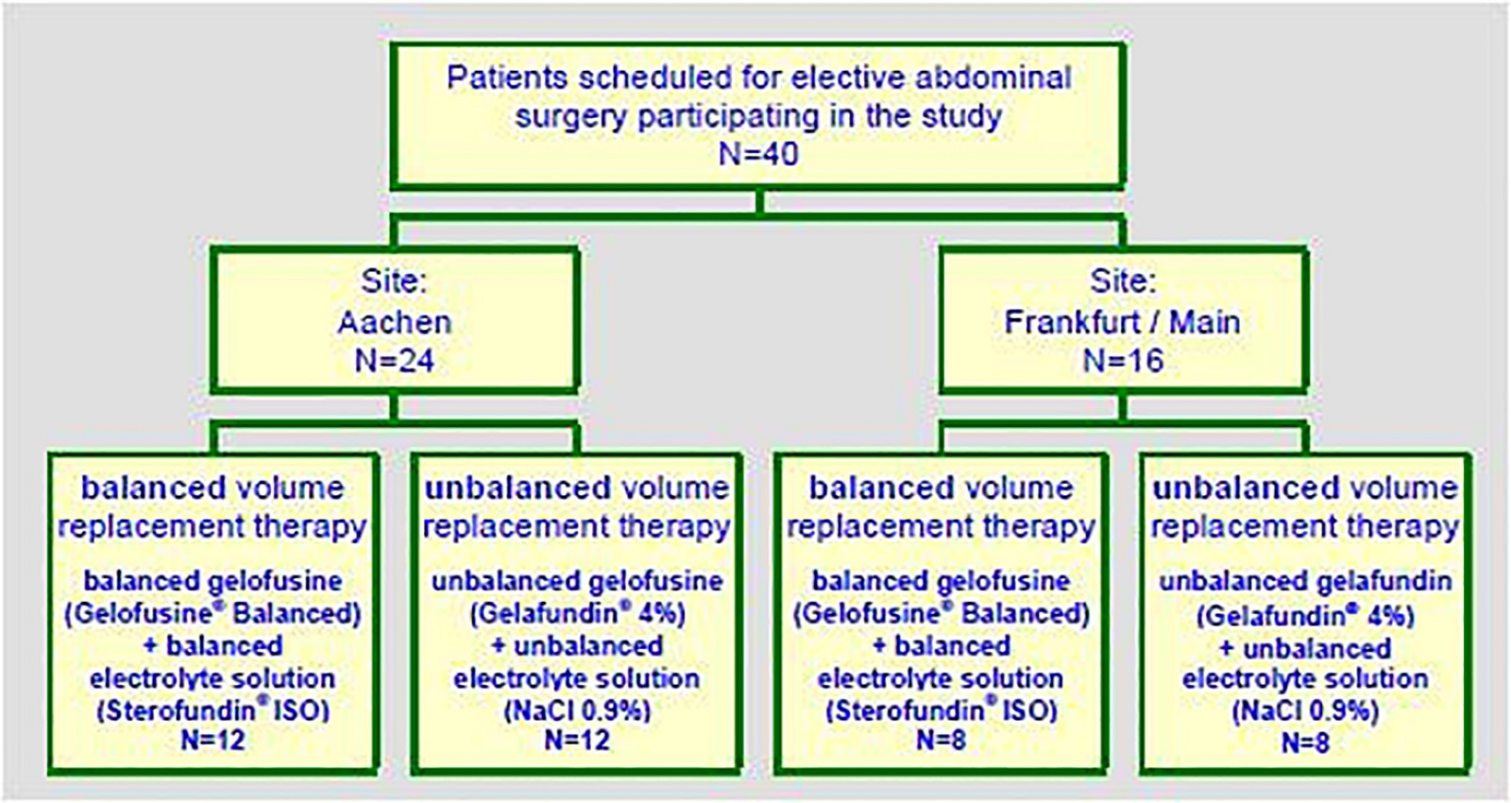
Study flow chart.

Randomisation of patients to either treatment group was performed in a 1:1 ratio. Patients were recruited between December 2011 and August 2012 in two tertiary care university hospitals. The study population covered male or female patients aged 18 to 90 years scheduled to undergo elective abdominal surgery with assumed intraoperative volume requirement of at least 15 mL/kg body weight gelatine solution. The aim was to study 50% of the patients in each side with half of the patients treated with the balanced regimen and the other half with the unbalanced regimen, each. The study flow chart is shown in Fig 1.

Standard operating procedures were in place for induction and maintenance of anaesthesia. Arterial and central venous lines were inserted prior to the induction of anaesthesia. Anesthesia included a standardized protocol of a balanced anesthesia using propofol and sevoflurane as volatile anesthetic in combination with a thoracic peridural anesthesia starting with 0.75% ropivacain, followed by a mixture of ropivacain 0.16% and sufentanil 0.5 μg/ml using an infusion pump and fentanyl as an opioid if needed. Standard measures of general critical care were applied to all patients including ventilation, analgosedation and weaning from the ventilator. Administration of the volume solutions was performed intravenously according to the patients’ need. The trigger for volume replacement was a CVP minus PEEP of less than 10 mmHg (CVP minus PEEP<10 mmHg). The crystalloid:colloid ratio was 1:1 intra- and postoperatively. We aimed at targets for volume replacement a CVP minus PEEP between 10 and 14 mmHg after treatment with vasoactive agent and MAP > 65 mmHg. The trigger for administration of red blood cells (RBCs) was a haemoglobin (Hb) ≤ 6 g/dl or Hb between 6–8 g/dl, in case of restricted compensation (e.g. coronary heart diseases, cardiac insufficiency and cerebrovascular insufficiency) and / or anaemic hypoxia (e.g. tachycardia, hypotension, ischaemia and lactate acidosis). The trigger for administration of fresh frozen plasma was acute bleeding or activated partial prothrombin time (aPTT) > 60s or fibrinogen < 2g/dl. For the administration of platelets two triggers were defined: bleeding or a platelet count < 50.000 / μl.

### Ethics committee

The protocol was approved by the local ethics committee of the University Hospital of RWTH Aachen (Aachen, Germany; Protocol No. HC-G-H-0904, Chairperson Prof. G. Schmalzing, date of approval: 22.08.2011). The approved recruitment period was between October 2011 and December 2012; the first patient was included in december 2011, the last patient was completed at August 31^th^ 2012. An informed consent, written in accordance with the Declaration of Helsinki and the applicable laws of the country was obtained from all patients.

The patient signed the Informed Consent Form before s/he entered the study, i.e. before screening bloods, other screening assessments or any other study-related activity. The patient was given sufficient time (preferably one day in advance) to consider the study's implications before deciding whether to participate.

### Inclusion and exclusion criteria

The following inclusion criteria applied:

Male or female patients aged ≥18 years and ≤ 90 years; women of child bearing potential must test negative on standard pregnancy test (urine dipstick).

- Patients who are willing to give voluntary consent to participate in the study, following a full explanation of the nature and purpose of the study, by signing the informed consent form approved by the Institutional Ethics Committee (IEC) prior to all evaluations.
- Patients scheduled to undergo elective major abdominal surgery (e.g. rectum resection, gastrectomy)
- Expected intraoperative volume requirement of at least 15 ml/kg body weight gelatine solution

Patients were not considered for participation in the study if any of the following exclusion criteria listed below applied:

- Patients of ASA-class > III
- Patients suffering from known hypersensitivity to gelatine or to any of the constituents of the gelatine solutions
- Patients suffering from hypervolaemia, hyperhydration, severe heart insufficiency or severe blood coagulation disorders, hypernatriaemia or hyperchloraemia, metabolic alcalosis, severe generalized oedema, intracranial haemorrhage, hyperkalaemia
- Estimated perioperative need for blood products of more than 3.5 mL/kg body weight
- Lactation period
- Simultaneous participation in another clinical trial
- Emergencies
- Patients suffering from
- moderate heart or lung insufficiency
- moderate lung oedema
- hypertonia
- eclampsia
- which do not allow the investigational product regimen as required.

### Data acquisition

Data were recorded preoperatively, at randomisation, prior and after induction of anesthesia until 12 hours after end of surgery including arterial blood gases. Arterial blood gas analyses were measured including BE, Hb, electrolytes and lactate. For renal function creatinine, creatinine clearance, urea nitrogen (BUN) and cystatin C were assessed using blood samples as well as diuresis, N-acetyl-beta-glucosaminidase (ß-NAG) and neutrophil gelatinase-associated lipocalin (NGAL) using urine samples. During surgery the administration of investigational products, surgery related data (e.g. start of intervention), haemodynamics, blood product administration, time on ventilation and adverse events / reactions were recorded. At the end of surgery drainage loss / estimated blood loss was documented. After admission to the intensive care unit (ICU) or intermediate care (IMC) and 6 hours after end of surgery drug monitoring and tolerance as well as episodes of secondary bleeding, concomitant medication and therapy, haemodynamics, time on ventilation, adverse events / reactions, length of stay (ICU / IMC) were recorded. 12 hours after end of surgery or at ICU / IMC discharge (which occurred latest) the last measurements took place. Thereafter volume replacement was performed at the discretion of the attending physician.

### Statistics

Changes from baseline to end of surgery in BE and chloride were the primary efficacy criteria. The sample size was calculated using Cohen’s *d* for changes in both chloride concentration and base excess with effect sizes of 1,711 and -0,910, respectively. The endpoints were compared in a non-prespecified order between the balanced gelatine regimen and the non-balanced regimen on the significance level of α = 0.025, two-sided and with a power (1-ß) of 0.80. Taking in to account a dropout rate of 10%, 64 patients were planned. Furthermore, a recalculation was planned after 40 patients, because acid base effects of balanced gelatin were unknown. This recalculation revealed that 16 patients per group were sufficient. Bonferroni-Holm was used to adjust for multiple testing.

All programming of tables, figures, listings, and statistical analyses were performed using the statistical software package SAS version 9.3 (TS1M2). After preparation of figures with SAS, the graphical presentations were produced using Graphpad Prism 6. All 'first level' baseline characteristics were evaluated by means of exploratory test statistics to check for balance between treatment groups (hierarchically subordinated parameters were in general not checked for homogeneity). For continuous measures, the U-test was used to analyze group differences. For categorical measures, χ2 test was used. The level α = 0.15 was used as significance level of homogeneity tests. The comparison of changes from baseline in BE and chloride between both groups was performed by means of ANCOVA with SITE as cofactor. The analysis was performed by means of non-parametrical methods as planned in the protocol and confirmed by parametric tests. If no differences occurred, the further analysis was based on parametric tests.

Missing values were not imputed. In tables of continuous and categorical data the number of missing data (Nmiss) is reported. In frequency tables of categorical data a category 'missing data' is provided whenever there are data missing, but this category was not be included in the calculation of percentages except when it appears reasonable on occasion.

After closure of the data base and determination of the analysis populations (in a blinded Data Review Meeting) the study was unblinded.

### Safety criteria

Changes from baseline to end of surgery / end of study were main criteria of the safety laboratory variables. The cumulated requirement of blood products and the incidences of adverse events–stratified for adverse event characteristics and MedDRA preferred terms (PTs) and system organ classes (SOCs)–were further main safety criteria.

## Results

### Patient characteristics

Forty patients (20 in each group) were enrolled in this investigation during a 9-month period. The planned re-calculation of sample size resulted in a sample size less than 40 and therefore the study was terminated according to the study protocol. Only one premature study termination occurred in one patient of the balanced group due to a change of the surgery by the surgeon. The baseline characteristics are summarized in Table 1 and did not differ between the two groups. Further indicators of coagulation are presented in the supplementary material (S1 Table).

**Table 1:** Baseline characteristics.

	Balanced (n = 20)	Unbalanced (n = 20)
Age (years)	64.9 ± 11.9	61.8 ± 9.8
Gender (male/female)	50% / 50%	75% / 25%
Body mass index (kg/m^2^)	24.4 ± 5.0	26.1 ± 4.4
ASA classification		
I	5.0%	—
II Table	50.0%	50.0%
III	45.0%	50.0%
Prevalent diseases (n)	34	41
Renal	4	3
Cardiac	3	5
Nervous system	3	1
Metabolic and nutritional disorders	5	9
Neoplasms	5	6
Respiratory	4	5
Vascular	10	12
Blood gas analysis		
Base excess (mmol/L)	-0.97 ± 3.03	0.03 ± 3.00
Chloride (mmol/L)	107.4 ± 2.9	106.8 ± 4.9
Haemoglobin (g/dL)	12.1 ± 1.8	13.4 ± 1.4
Haematocrit (%)	37.3 ± 5.5	41.3 ± 4.3
Haemodynamics		
Systolic arterial pressure (mmHg)	134 ± 16	134 ± 17
Diastolic arterial pressure (mmHg)	72 ± 11	75 ± 12
Mean arterial pressure (mmHg)	93 ± 11	95 ± 12
Heart rate (beats/min)	82 ± 14	79 ± 11
Central venous pressure (mmHg)	11 ± 3	10 ± 6
Positive end-expiratory pressure (cmH_2_O)	5 ± 2	4 ± 2
Renal function		
Crea Cl (ml/min)	90±30	100±38
S_crea_ (mg/dl)	0.8±0.4	0.9±0.3
BUN (mg/ml)	26±17	31±18
Cystatin C (mg/L)	0.90±0.22	1.00±0.33
ß-NAG (U/L)	7.8±7.2	12.1±18.8
NGAL (ng/mL)	23±56	25±24
Coagulation		
AT III	90.4±15.0	87.9±15.3
aPTT (sec)	29.8±4.7	31.4±5.6
Platelet count (/nL)	287±92	274±122
Fibrinogen (g/L)	3.5±0.7	3.7±1.2
ROTEM		
Ex-TEM: CT (sec)	58±13	62±14
Ex-TEM: MCF (mm)	67±7	67±7
In-TEM: CT (sec)	175±26	161±37
In-TEM: MCF (mm)	66±7	66±9

All data are presented as mean ± SD unless otherwise indicated. AT III = Antithrombin III; aPTT = activated partial thromboplastin time; ASA = American Society of Anesthesiology; ß-NAG = N-acetyl-ß-D-glucosaminidase; BUN = blood urea nitrogen; Crea Cl = Creatinine Clearance; CT = Clotting time; Ex-TEM = ROTEM activated by tissue factor (extrinsic pathway of coagulation); In-TEM = ROTEM activated by phospholipid and ellagic acid (intrinsic pathway of coagulation); MCF = maximum clot firmness; NGAL = neutrophil gelatinas-associated lipocalin; ROTEM = Thrombelastometry; S_crea_ = Serum-Creatinine.

There were no statistical differences between the groups at baseline

### Blood gas analysis:

**Fig 2:**
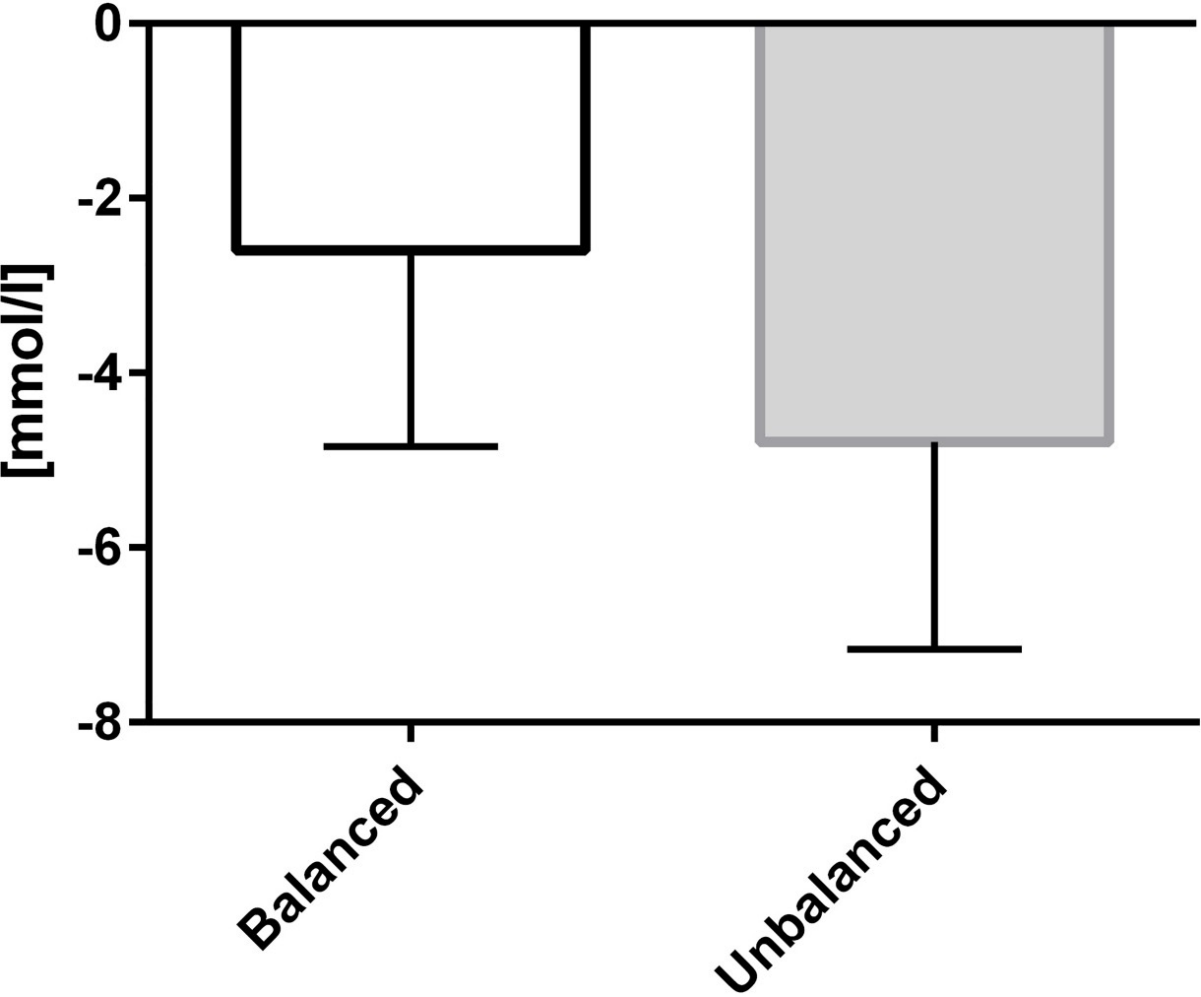
Change of base excess from baseline until the end of surgery. Data presented as mean ± SD; p = 0.0117.

**Fig 3:**
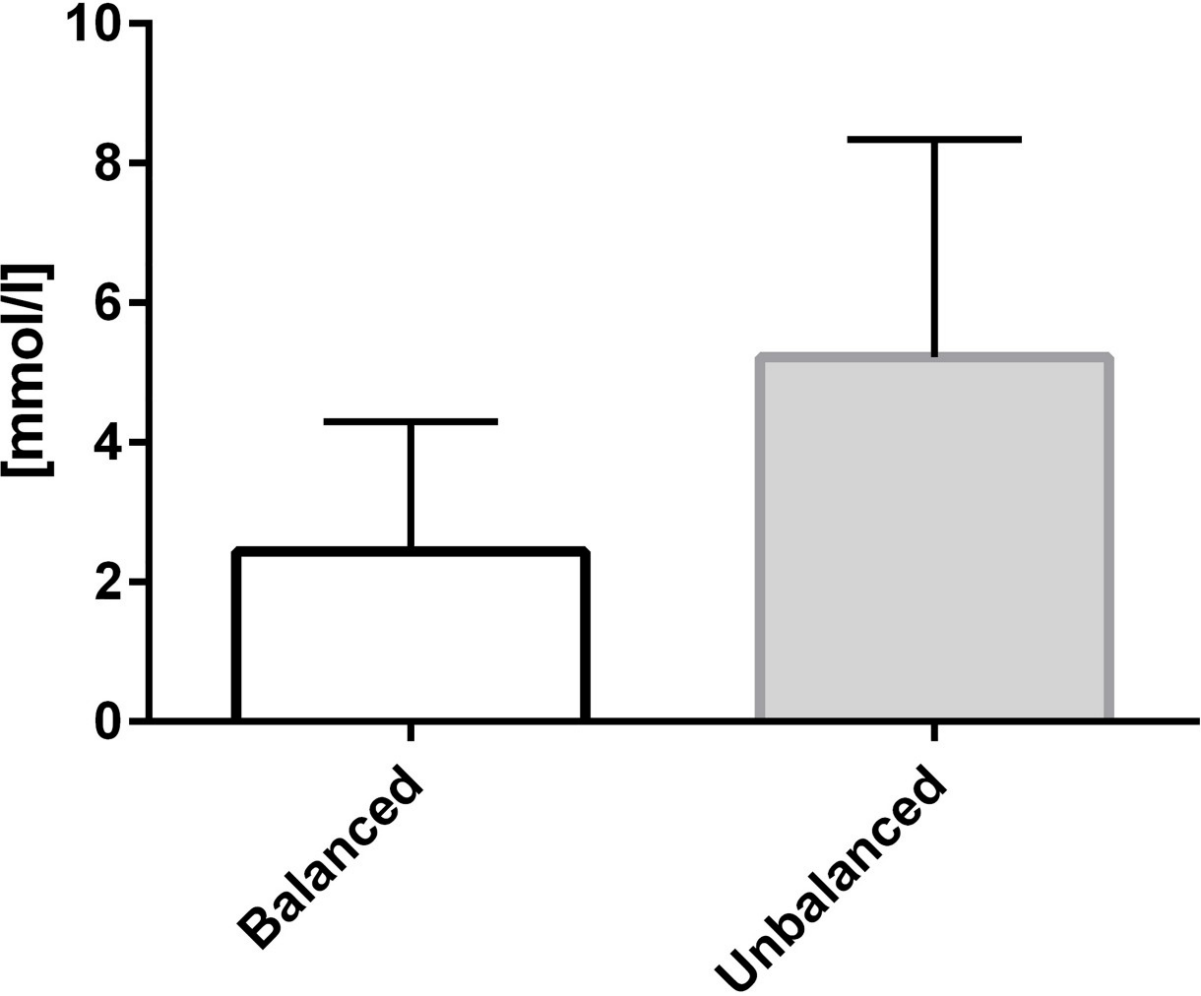
Change of serum chloride from baseline until the end of surgery. Data presented as mean ± SD; p = 0.0045.

The primary endpoints, intraoperative changes of base excess and chloride, resulted in statistically significant differences (p = 0.0117, p = 0.0045 respectively) as displayed in Figs 2 and 3. The change in base excess resulted in –2.59 ± 2.25 (median: –2.65) mmol/L (balanced group) and –4.79 ± 2.38 (median: –4.70) mmol/L (non-balanced group). Regarding chloride the results were 2.4 ± 1.9 (median: 3.0) mmol/L and 5.2 ± 3.1 (median: 5.0) mmol/L, respectively. Bicarbonate decreased by -2.31±2.32 mmol/L in the balanced group and by -4.52±2.15 mmol/L in the unbalanced group. An alpha-adaption according to Bonferroni-Holm confirmed the superiority of the balanced treatment for both primary endpoints and bicarbonate. Changes in pH and pCO_2_ did not differ significantly between the groups.

The postoperative changes from baseline of base excess and chloride were in favor of the balanced group (p = 0.0044). While the base excess increased by 3.35 ± 1.64 (median –3.50) mmol/L in the balanced group during the IMC/ICU stay and did not change further until the end of the study, it decreased significantly by –0.58 ± 3.14 (median –1.15 mmol/L) from baseline in the non-balanced group. The chloride levels increased significantly more in the non-balanced group, 6.6 ± 4.5 (median 6.0) mmol/L versus 2.0 ± 2.9 (median 2.0) mmol/L.

### Renal function

In both study groups, no single indicator of renal function differed significantly between the groups over the study period. The urinary excretion during the study period was 1,482±481 ml and 1,594±530 ml in the balanced and unbalanced group, respectively. Creatinine, creatinine clearance, BUN, Cystatin C, ß-NAG, and NGAL varied slightly over the study period, but the changes were not significantly different between the groups; neither during nor after the operation (Table 2).

**Table 2:** Renal function and coagulation: Changes during the intra- and postoperative period.

	Balanced (n = 19)		Unbalanced (n = 20)	
Parameter	Intraoperative	Postoperative	Intraoperative	Postoperative
Renal function				
S_crea_ (mg/dL)	-0.04±0.15	-0.02±0.21	-0.04±0.14	-0.01±0.14
Creatinine Clearance (mL/min)	5.7±17.2	5.2±26.6	5.6±12.2	2.97±12.45
BUN (mg/dL)	1.9±5.3	5.3±7.6	-0.3±5.2	1.7±9.5
Cystatin C (mg/ml)	-0.11±0.15	-0.05±0.17	-0.16±0.17	-0.10±0.14
ß-NAG (U/L)	2.6±12.8	-0.7±10.6	-1.0±9.6	-5.4±13.8
NGAL (ng/mL)	24.5±38.5	13.7±34.4	19.4±39.0	27.1±107.1
Coagulation				
AT III	-28.4±11.5	-30.4±14.0	-25.7±12.6	-26.3±10.6
aPTT (sec)	0.1±4.4	4.8±5.6	-2.0±7.7	6.9±22.6
Platelet count (/nL)	-59.0±50.1	-71.1±55.1	-59.6±60.2	-82.7±79.1
Fibrinogen (g/L)	-1.1±0.5	-0.6±0.5	-1.4±1.0	-1.0±1.2
ROTEM				
Ex-TEM: CT (sec)	4.1±18.2	5.1±10.8	-3.7±21.5	1.9±15.2
Ex-TEM: MCF (mm)	-5.7±6.2	-6.4±8.3	-5.6±8.5	-6.4±8.3
In-TEM: CT (sec)	-13.1±32.2	12.6±29.8	-0.4±51.2	32.7±115.1
In-TEM: MCF (mm)	-6.1±9.9	-5.9±6.7	-4.9±4.4	-5.8±4.5

All data are presented as mean±SD unless otherwise indicated. AT III = Antithrombin III; aPTT = activated partial thromboplastin time; ß-NAG = N-acetyl-ß-D-glucosaminidase; BUN = blood urea nitrogen; Crea Cl = Creatinine Clearance; CT = Clotting time; Ex-TEM = ROTEM activated by tissue factor (extrinsic pathway of coagulation); In-TEM = ROTEM activated by phospholipid and ellagic acid (intrinsic pathway of coagulation); MCF = maximum clot firmness; S_crea_ = Serum-Creatinine

P-values were calculated with the U test. None of the changes were significantly different between the groups.

### Coagulation

The coagulation tests did not differ significantly between the groups during the operation. Starting from a higher baseline value in the balanced group (608±407 U vs. 284±298 U; p = 0.0995) the changes in ADP-induced thrombocyte aggregation differed significantly between the balanced compared to the unbalanced group (-228±149 U vs. 105±393 U). However, the threshold for compromised platelet function is <45 U, thus neither balanced nor unbalanced solutions had a relevant influence on coagulation. Further data on coagulation are given in Table 2 and S2 Table.

### Volume therapy, use of blood products and haemodynamics

In both groups the investigational product administration in terms of volume and infusion rate was comparable throughout the course of the study, i.e. before, during and after surgery. Nine patients (three in the balanced and six in the non-balanced group) received blood products intraoperatively as presented in Table 3.

**Table 3:** Applied investigational crystalloids and gelatine solutions (volume and infusion rate) and blood products.

		Balanced		Unbalanced	
Volume therapy		n = 20 [ml]	n = 20 [ml/kg/h]	n = 20 [ml]	n = 20 [ml/kg/h]
Crystalloid	Before surgery	440 ± 176	8.6 ± 4.2	501 ± 137	10.0 ± 4.5
	During surgery	1354 ± 714	6.3 ± 2.9	1519 ± 1025	7.0 ± 2.8
	After surgery	1888 ± 804	2.4 ± 0.8	1782 ± 919	3.1 ± 2.0
Gelatine solution	Before surgery	167 ± 156	9.3 ± 4.9	151 ± 158	8.4 ± 3.2
	During surgery	1514 ± 727	7.2 ± 3.0	1619 ± 725	7.8 ± 3.1
	After surgery	1078 ± 720	8.1 ± 2.8	1094 ± 734	9.4 ± 5.9
Blood products		n = 3		n = 6	
Fresh frozen plasma (ml)		750 ± 0		1188 ± 554	
Red blood cells (ml)		750 ± 661		875 ± 494	
Blood products total (ml)		1000 ± 1090		1667 ± 1201	

All data are presented as mean ± SD unless otherwise indicated.

There were no significant differences between the groups.

The haemodynamics (SAP, DAP, MAP, CVP) did not show any treatment differences, except from CVP which decreased suring surgery in the balanced group by -3.5±6.1 mmHg while it increased by 1.2±7.1 mmHg in the unbalanced group. In both groups a decrease during surgery and a clear increase up to the end of surgery was observed. The haemodynamic values are shown in the supplementary material (S2 Table).

### Surgeries and mechanical ventilation

Out of the performed surgeries the most frequent were cholecystectomy, hepatectomy, pancreaticoduodenectomy, adhesiolysis, colectomy, intestinal anastomosis, pancreatectomy and resection of rectum. The duration of surgery defined as time from incision to wound suture did not differ between the balanced and non-balanced group (4.25 ± 1.21 h and 4.26 ± 1.79 h, p = 0.8993). The time of intubation was comparable between both groups (5.97 ± 2.84 h–balanced group versus 6.63 ± 3.30 h–non-balanced group). After end of surgery, however, 9 patients (47%) of the non-balanced group compared to 5 patients (26%) of the balanced group needed mechanical ventilation after surgery. The duration of ICU / IMC stay was comparable between the two groups.

### Adverse events / reactions

For almost all patients of both groups at least one adverse event (no relation to investigational product) was documented for the time after first application of investigational product. All events were neither serious nor of severe intensity. The most often documented events belong to the system organ class (SOC) “investigations” like increase of chloride. One patient suffered from a serious adverse event after admission to the intensive care unit. An increase of creatine phosphokinase was assessed as probably related to an ischaemia of both legs due to a known peripheral artery occlusive disease.

The occurrence of adverse reactions (at least possibly related to investigational product administration) was comparable between the two groups. In each group 15 patients suffered from an adverse reaction. None of the reactions was serious or of a severe intensity. The most frequent reactions according to system organ classes (SOCs) were “investigations”, “metabolism and nutrition disorders” and “blood and lymphatic system disorders” detected by laboratory values.

## Discussion

This prospective, controlled, randomised, double-blind, dual centre study demonstrated safety using the balanced gelatine solution Gelaspan 4% combined with Sterofundin ISO perioperatively in surgical patients. In addition, significantly smaller influences on blood gas analytic parameters, in BE and chloride, in comparison with the unbalanced gelatine solution Gelafundin 4% combined with NaCl 0.9% could be shown. To our knowledge this is the first prospective randomized clinical study investigating the effect of a balanced colloid–here gelatin–and a balanced crystalloid vs. unbalanced solutions.

Data from a large 7 day observational trial (European Surgical Outcome Study) including more than 46.000 surgical patients showed, that the mortality after surgery excluding neuro- and cardiothoracic surgery is as high as 4% across Europe.[8] That implies an urgent demand to improve perioperative care. One way to improve post-operative outcome is the introduction of perioperative haemodynamic optimization (PHO).[9] Several meta-analysis have confirmed the ability of PHO to reduce the rate of post-operative acute kidney injury,[10] gastro-intestinal complications,[11] pneumonia, surgical site and urinary tract infections,[12] and the rate of patients developing at least one post-operative complication.[13] The majority of all these studies included a protocol based treatment including colloids and inotropic support if indicated by flow based variables, which in turn are a strong marker for the use of colloids like gelatine, if indicated.

The volume expanding effect of gelatine compared to HES has been a matter of debate. However, Lobo and coworkers could show in volunteers that 4% succinylated gelatine increased blood volume to a similar extent as 6% HES 130/0.4 and better than 0.9% NaCl.[14] NaCl 0.9% also increased the extravascular compartment significantly more compared to 4% succinylated gelatine and 6% HES 130/0.4. Similar results were demonstrated by the same group comparing 4% gelatine versus 6% HES 130/0.4 in 25 adult patients undergoing surgery [15].

Yates et al. performed recently an randomized single center study in 202 high risk surgical patients undergoing colorectal surgery comparing effects of balanced 6% HES 130 versus a balanced crystalloid on hemodynamic stability and organ function.[16] The primary objective was the gastrointestinal morbidity on the 5^th^ postoperative day. The authors found that HES did not maintain the splanchnic circulation more effectively, but also no actual coagulopathy was induced using HES. No increase in thrombotic events was seen in the crystalloid group. The authors concluded that despite HES patients requiring statistically significant less fluid than those in the crystalloid group (5.3 liters versus 6.3 liters), there was no associated clinical benefit. Interestingly, in 38% of the crystalloid group the use of gelatine as the predefined rescue fluid was necessary to achieve the predefined hemodynamic goals. This was only true for 12% of patients in the HES group. This difference was statistically significant. This result supports the position that in high risk surgical patients gelatine is as effective as HES to achieve perioperative hemodynamic optimization.[17]

Our results are in line with the positive evidence on the use of balanced fluid replacement in the OR and the ICU environment. It is known that infusion of large amounts of colloids in an unbalanced solution like 0.9% NaCl, leads to discrete disturbances in acid-base balance [5]. BE may serve as an important marker to identify patients with under-perfused tissue. Acidosis produced by volume replacement of non-balanced solutions may mask the diagnosis of perfusion deficits or may result in inappropriate clinical interventions due to the erroneous presumption of ongoing tissue hypoxia secondary to hypovolaemia. The BE ‘removes’ the respiratory element of acid-base disturbance and a metabolic acidosis can be diagnosed.[18] Thus, BE besides chloride was chosen as primary variable in this study comparing balanced and non-balanced solutions. Feldheiser et al. investigated in a randomized study 50 patients undergoing elective ovariectomies.[19] The authors looked at the effects of balanced 6% HES 130 versus balanced crystalloid up to 50ml/kg on optimizing haemodynamics and preventing organ dysfunction. A Doppler device was used to achieve the predefined goal-directed therapy. The authors could demonstrate no differences in intraoperative heart rate, mean arterial or central venous pressure, or norepinephrine requirements, but the use of the balanced HES solution was associated with higher stroke volume, cardiac index and corrected flow time, hence better haemodynamic stability. In addition, they showed a reduced need for fresh-frozen plasma in the HES group. Furthermore, there were no signs of renal impairment by balanced colloid compared to a balanced crystalloid in this trial. Mahmood et al. demonstrated that the intraoperative use of gelatine versus two different HES solutions in 62 patients undergoing elective abdominal aortic aneurysm (AAA) repair was associated with more renal dysfunction.[20] Renal function was assessed by measuring creatinine, urinary α1-microglobulin:creatinine ratio and urinary immunoglobulin G:creatinine. It might be that the use of non-balanced gelatine solutions could explain the differences to the results of the present study. Moreover, a Cochrane analysis demonstrated that the perioperative use of balanced compared to the use of unbalanced fluids is equally safe and effective.[21] In addition, the use of balanced fluids are associated with less metabolic derangement, less hyperchloraemia and less metabolic acidosis emphasizing the importance of chloride on renal function and acid base status.[21] Yunos showed in two studies that in ICU patients a balanced based fluid regimen[22, 23] resulted into significant less acute kidney injury and less requirement of renal replacement therapy compared to a chloride based fluid regimen. In contrast, Young et al. could recently not find a difference in ICU patients comparing balanced crystalloid solution versus 0.9% NaCl solution.[24] It has to be noticed that in this study the vast majority of the included patients were not critically ill, had a short ICU stay and received only 2000mL of study fluid.

In a recently published evidenced-based guideline the use of balanced solutions, crystalloids as well as colloids was recommended with a grade A recommendation in the OR and in the ICU environment.[25]

Several limitations within our study have to be addressed. First of all, we have investigated only a small number of patients, i.e. 40 patients. However, this number of patients was included according to our sample size calculation and power analysis, and sufficient in order to create meaningful data, which is reflected by the obtained statistical significant results. In addition, we observed the patients only during a short period of time, i.e. until 12 hours after end of surgery. We did not observe any allergic reaction to gelatine infusion. This might also be due to the small number of patients. On the other hand, in two large studies investigating the allergic risk of volume replacement solutions there was a higher allergic risk associated only with the use of urea-linked gelatine infusion,[26] whereas the allergic risk of modified gelatine infusion was comparable with HES or albumin. We measured the CVP as trigger and target value. According to latest NICE recommendations flow based variables would have been preferable, although the use of CVP is still common practice in Germany in the OR and on the ICU.

## Conclusion

In conclusion, this study showed that a balanced gelatine solution compared with an unbalanced solution reduced acid-base-imbalances when used in the perioperative care of surgical patients in the OR and up to 12 hours postoperatively. Renal function and coagulation test remained similar with both solutions.

We could demonstrate significantly smaller influences using balanced gelatine solution combined with a balanced electrolyte solution on blood gas analytic parameters, in BE and serum chloride, in comparison with the non-balanced 4%gelatine solution combined with NaCl 0.9%.

No marked treatment differences between the groups were observed with respect to haemodynamics, coagulation, and renal function. Thus, in this study the use of balanced gelatine in the perioperative situation showed no potential to harm and was associated with beneficial effects on the metabolic situation.

## Supporting information

S1 File CONSORT checklist. (DOC)

S2 File Study protocol–Redacted. (PDF)

S1 Table Additional markers of coagulation. (DOCX)

S2 Table Haemodynamics. (DOCX)
