# Supplementary material for: Impact of a new balanced gelatine on electrolytes and pH in the perioperative care
Source: PLoS One. 2019 Apr 29;14(4):e0213057. doi: 10.1371/journal.pone.0213057 (PMC6488052; doi:10.1371/journal.pone.0213057)
Supplement: S2 File — (PDF) [file pone.0213057.s002.pdf]

CLINICAL STUDY PROTOCOL

PROSPECTIVE CONTROLLED DOUBLE-BLIND PHASE III BICENTER STUDY ON THE EFFICACY AND SAFETY OF A BALANCED GELATINE SOLUTION IN COMBINATION WITH A BALANCED ELECTROLYTE SOLUTION VERSUS A STANDARD GELATINE SOLUTION IN COMBINATION WITH A NON-BALANCED ELECTROLYTE SOLUTION IN PATIENTS SCHEDULED FOR ABDOMINAL SURGERY

STUDY IDENTIFICATION NO.: HC-G-H-0904
EUDRACT NO.: 2010-018524-58

DIVISION HOSPITAL CARE
B. BRAUN MELSUNGEN AG
CARL-BRAUN-STR. 1
34212 MELSUNGEN
GERMANY

Final version xx	2010-10-06
Final version 02 incorporating Amendment/s No. 1, dated 2011-07-25	2011-07-25
Final version 03 incorporating Amendment/s No. 1, dated 2011-07-25 and No. 2 dated 2011-12-12	2011-12-12
Final version 04 incorporating Amendment/s No. 1, dated 2011-07-25, No. 2 dated 2011-12-12 and No. 3 dated 2012-04-17	2012-05-08

All information in this study protocol including attachments provided to you as investigator, potential investigator, co-investigator or adviser must be treated confidential. The right to use this information is limited to you, your staff, members of the IRB or entitled authorities. The objectives and content of this study, as well as its results, must be treated confidential and may not be made available to third parties at any time before, during and after the study without written approval of the B. Braun Group except to the extent necessary to get informed consent from patients. This applies to investigators and all supporting staff involved in the study. Transmission, duplication or use for publication is permitted only with the written agreement of the B. Braun Group.

This protocol has been written in accordance with current ICH-GCP guidelines.

TABLE OF CONTENTS

1 RESPONSIBILITIES AND ADDRESSES 5

2 ABBREVIATIONS AND DEFINITIONS 8

3 PROTOCOL SUMMARY / SYNOPSIS 10

4 INTRODUCTION 19

4.1 BACKGROUND INFORMATION 19
4.2 STUDY RATIONALE 20
4.3 RISK-BENEFIT-ASSESSMENT 21

5 STUDY OBJECTIVES 21

5.1 PRIMARY OBJECTIVE AND PRIMARY VARIABLE(S) 21
5.1.1 Primary Objective 21
5.1.2 Primary Variable 21
5.2 SECONDARY OBJECTIVES AND SECONDARY VARIABLES 22

6 STUDY DESIGN 22

7 SELECTION AND WITHDRAWAL OF PATIENTS 23

7.1 INFORMED CONSENT 23
7.2 PATIENT INCLUSION CRITERIA 23
7.3 PATIENT EXCLUSION CRITERIA 23
7.4 STOPPING AND DISCONTINUATION CRITERIA 24
7.4.1 Discontinuation Criteria Related to the Study 24
7.4.2 Discontinuation Criteria Related to the Study Site 25
7.4.3 Discontinuation Criteria related to the Patient 25
7.5 RANDOMIZATION, BLINDING AND UNBLINDING 26

8 INVESTIGATIONAL PRODUCTS 27

8.1 NAME AND DESCRIPTION OF THE INVESTIGATIONAL PRODUCT(S) 27
8.1.1 Qualitative and Quantitative Composition 27
8.1.2 Pharmaceutical Form 28
8.1.3 Nature and Content of Container(s) 28
8.2 POSOLOGY AND METHOD OF ADMINISTRATION 28
8.2.1 Dosage 28
8.2.2 Method of Administration 29
8.2.3 Duration of treatment 29
8.3 LABELLING 29
8.4 PACKAGING 32
8.5 STORAGE 32
8.6 INVESTIGATIONAL PRODUCT ACCOUNTABILITY 32
8.7 DESTRUCTION / RETRIEVAL OF SURPLUS INVESTIGATIONAL PRODUCTS 33

9 CONCOMITANT THERAPY 33

10 DEFINITION OF THE PRIMARY AND SECONDARY VARIABLES 34

10.1 PRIMARY VARIABLE(S) 34
10.2 SECONDARY VARIABLES 34
10.2.1 Secondary Variables – Safety 34
10.2.2 Secondary Variables - Efficacy 37
10.3 OTHER VARIABLES 38
10.4 SOURCE DOCUMENTS 39

11 ASSESSING AND REPORTING OF ADVERSE EVENTS 40

11.1 DEFINITIONS 40
11.1.1 Adverse Events 40
11.1.2 Adverse Reaction 40
11.1.3 Unexpected Adverse Reactions 40

11.1.4 Serious Adverse Event (SAE) or Serious Adverse Reaction (SAR)......41
11.1.5 Adverse Event Intensity......41
11.1.6 Adverse Event Causality......42
11.2 RECORDING AND REPORTING ADVERSE EVENTS AND ADVERSE REACTIONS......42
11.2.1 Recording......42
11.2.2 Reporting of Serious Adverse Events and Unexpected Adverse Reactions......43
11.3 ADVERSE EVENT FOLLOW-UP PROCEDURES......43
11.4 POTENTIAL RISKS AND POTENTIAL ADVERSE EVENTS......43
11.5 PREGNANCIES......45

12 STUDY SCHEDULE......45

12.1 PROCEDURES AT EACH VISIT......45
12.2 TABULAR OVERVIEW......48
12.3 ASSESSMENT OF COMPLIANCE......50
12.4 PRECAUTIONARY MEASURES......50

13 STUDY AND TREATMENT DURATION......52

13.1 DURATION PER PATIENT......52
13.2 DURATION OF WHOLE STUDY......52

14 STATISTICS......52

14.1 STATISTICAL METHODS......52
14.2 INTERIM ANALYSIS......53
14.3 LEVEL OF SIGNIFICANCE AND POWER......53
14.4 STATISTICAL HYPOTHESES......53
14.5 SAMPLE SIZE......54
14.6 DATA HANDLING......54
14.7 CRITERIA FOR THE TERMINATION OF THE STUDY......55
14.8 PATIENT SELECTION FOR ANALYSES......55

15 SOURCE DATA AND SOURCE DOCUMENTS......55

15.1 DEFINITIONS......55
15.1.1 Source Documents......55
15.1.2 Source Data......56
15.1.3 Direct Access......56
15.2 PERMISSION OF ACCESS......56

16 QUALITY CONTROL AND QUALITY ASSURANCE......56

16.1 QUALITY CONTROL......56
16.1.1 Definition......56
16.1.2 Study Monitoring......56
16.2 QUALITY ASSURANCE......57
16.2.1 Definition......57
16.2.2 Audit......57
16.2.3 Inspection......57

17 ETHICAL AND LEGAL CONSIDERATIONS......57

17.1 COMMITTEES AND BOARDS......57
17.1.1 Independent Ethics Committee (IEC)......57
17.1.2 Institutional Review Board (IRB)......58
17.1.3 Drug Safety Monitoring Board (DSMB)......58
17.2 CONDUCT OF STUDY AND ETHICAL CONSIDERATIONS......58
17.3 RESPONSIBILITIES......58
17.4 GENERAL REPORTING OBLIGATION......59
17.5 FINANCING AND INSURANCE......59
17.6 PERSONAL DATA AND DATA PROTECTION......59
17.7 MODIFICATION OF PROTOCOL......59
17.8 INVESTIGATOR'S BROCHURE/SUMMARY OF PRODUCT CHARACTERISTICS......60
17.9 COMPLETION OF CASE REPORT FORMS......60
17.10 ARCHIVING......60
17.11 CONFIDENTIALITY......60

18 FINAL REPORT AND PUBLICATION POLICY 60

19 REFERENCES 62

20 SIGNATURES 63

21 APPENDICES .... **FEHLER! TEXTMARKE NICHT DEFINIERT.**

1 Declaration of Helsinki (version 1996)

2 Serious Adverse Event Form

3 Laboratory Methods

4 Reference Ranges

1 Responsibilities and addresses

Coordinating Investigator

Principal Investigator / Aachen

Investigator(s) / Aachen

Principal Investigator / Frankfurt am Main

Investigator(s) / Frankfurt am Main

Sponsor

B. Braun Melsungen AG
Carl-Braun-Str. 1
34212 Melsungen - Germany

Sponsor's representative

Medical representative of the Sponsor

Study manager

Statistician (Planning)

SAE contact details
Emergency contacts

CRO/Monitor

Laboratory

Pharmacy:

2 ABBREVIATIONS AND DEFINITIONS

aPTT	Activated Partial Thrombin Time
ABS	Acid Base Status
ADP	Adenosine Diphosphate
AE	Adverse Event
ANOVA	Analysis of Variance
ASA	American Society of Anesthesiologists
AR	Adverse (Drug) Reaction
ASPI	Arachidonic Acid
ß-NAG	*N*-Acetyl-*β*-Glucosaminidase
BE	Base Excess
BUN	Blood Urea Nitrogen
Broca	Normal weight [kg] = Height [cm] – 100, according to the formula of Pierre Paul Broca
CLI	Clot Lysis Index
CRF	Case Report Form
CRO	Contract Research Organization
CT	Clotting Time
CTM	Clinical Trial Medication
CVP	Central Venous Pressure
DAP	Diastolic Arterial Pressure
DSMB	Drug Safety Monitoring Board
e.g.	Exempli Gratia, for example
FAS	Full Analysis Set
FFP	Fresh Frozen Plasma
GCP	Good Clinical Practice
GFR	Glomerular Filtration Rate
GMP	Good Manufacturing Practice
Hb	Hemoglobin
Hct	Hematocrit
HCO_3	Bicarbonate
HES	Hydroxyethylstarch
HR	Heart Rate
IB	Investigator’s Brochure
ICH	International Conference on Harmonization
ICU	Intensive Care Unit
ID	(Patient) Identification

IEC	Independent Ethics Committee
IMC	Intermediate Care
IP	Investigational Product
IRB	Institutional Review Board
ITT	Intent-To-Treat
MAP	Mean Arterial Pressure
MCF	Maximum Clot Firmness
MFG	Modified Fluid Gelatine
NaCl	Sodium Chloride
NA	Not Applicable
NGAL	Neutrophil Gelatinase Associated Lipocalin
pCO_2	Partial Pressure of Carbon Dioxide
Ph. Eur.	Pharmacopoea Europaea
PEEP	Positive End-Expiratory Pressure
pH	pondus Hydrogenii
pO_2	Partial Pressure of Oxygen
PC	Platelet Count
PP	Per Protocol
RBC	Red Blood Count
S_{Crea}	Serum Creatinine
SaO_2	Arterial Oxygen Saturation
SAE	Serious Adverse Event
SAP	Systolic Arterial Pressure
SAP	Statistical Analysis Plan
SAR	Serious Adverse Reaction
SmPC	Summary of Product Characteristics
SOP	Standard Operating Procedure
SUSAR	Suspected Unexpected Serious Adverse Reaction
TRAP	Thrombin Receptor Activating Peptide
VCAS	Valid Case Analysis Set

3 PROTOCOL SUMMARY / SYNOPSIS

Title of Study	Prospective controlled double-blind phase III bicentre study on the efficacy and safety of a balanced gelatine solution in combination with a balanced electrolyte solution versus a standard gelatine solution in combination with a non-balanced electrolyte solution in patients scheduled for abdominal surgery
Investigational Products	Investigational test product:<br>Gelofusine® Balanced<br>combined with Sterofundin® ISO<br><br>Investigational reference product:<br>Gelafundin® 4%<br>combinded with NaCl 0.9%
Phase	III
Study Design	Prospective, controlled, randomized, double-blind, bicentre study performed in two parallel groups
Number of Sites & Countries	2 sites in Germany
Sample Size	64 patients, i.e. 32 patients per group
Indication	Perioperative plasma volume replacement in abdominal surgery
Primary Objective	Investigating the change in acid-base-balance of the two Gelofusine® regimes from baseline to end of surgery
Primary Variable	Base excess (BE) and chloride
Secondary Objectives	Investigation of safety and efficacy parameters of the balanced Gelofusine® regimen in comparison to the non-balanced regimen.
Secondary Variables	Safety:<br>▪ Arterial blood gas analysis<br>o pH<br>o BE<br>o HCO_3<br>o Lactate<br>o Sodium<br>o Potassium<br>o Calcium<br>o Chloride<br>o pCO_2<br>o pO_2<br>o SaO_2<br>o Hb<br>o Hct<br><br>measurements: baseline (i.e. before induction of anesthesia), intraoperatively every 15 min including

	recovery room every hour and additional measurements depending on the investigators decision, admission ICU / IMC, 6 hours and 12 hours after end of surgery<br>▪ Coagulation status<br>o Antithrombin III<br>o Fibrinogen<br>o aPTT<br>o Platelet count<br>measurements: baseline, admission ICU / IMC, 6 hours and 12 hours after end of surgery<br><br>o ROTEM (CT, MCF, LI 30)<br>o Platelet aggregation (TRAPtest, ASPItest, ADPtest) – only site Frankfurt am Main<br>measurements: baseline, admission ICU / IMC, 6 hours and 12 hours after end of surgery<br>▪ Renal function<br>o Serum creatinine<br>o Creatinine clearance<br>o BUN<br>o Cystatin C<br>o Amount of urine excreted (diuresis)<br>o *N*-acetyl-beta-glucosaminidase (β-NAG)<br>o NGAL<br>measurements: baseline, admission ICU / IMC, 12 hours after end of surgery<br>o GFR to be calculated during statistical evaluation<br>▪ Requirement of blood products<br>o RBC<br>o FFP<br>o Other (e.g. thrombocyte concentrate)<br>measurement: perioperatively<br><br>o Drainage blood loss and estimated intraoperative blood loss<br>measurement: 12 hours after end of surgery<br>▪ Adverse events<br>measurements: continuously during study period<br><br>Efficacy:<br><br>▪ Hemodynamics<br>o SAP, DAP, MAP, HR, CVP, PEEP<br>measurements: baseline, intraoperatively every 15 min, end of surgery, admission to ICU / IMC and thereafter every 6 hours<br>▪ Investigational product administration according to the defined algorithm<br>o Start and end, amount given<br>measurements: during IP-administration<br>o Amount given intraoperatively (i.e. start of IP-administration until end of anesthesia)<br>o Amount given postoperatively (i.e. end of anesthesia until 12 hours after end of surgery)

	Clinical Outcome:<br>▪ Time on ventilator<br>measurement: from intubation to extubation in hours; the need of reintubation as well as requirements of postoperative ventilatory support will be documented<br>▪ Length of stay in ICU / IMC (i.e. time of discharge from ICU / IMC)<br>▪ Secondary bleeding<br>▪ Concomitant medication/ therapy<br>o Start, end, drug / active substance, dose and unit, indication<br>measurements: continuously during study period<br><br>Other<br>▪ Demographic data<br>o Age, height, weight, sex, ASA-classification, concomitant disease, diagnosis, Apfel-Score (preoperative)<br>▪ Surgery related data<br>o Kind of intervention<br>o Date of intervention<br>o Duration of anesthesia<br>o Duration of intervention
Patient Inclusion / Exclusion Criteria	Inclusion criteria:<br>▪ Male or female patients ≥ 18 years of age and ≤ 90 years of age. Women of child bearing potential must test negative on standard pregnancy test (urine dipsticks).<br>▪ Patients scheduled to undergo elective abdominal surgery (e.g. rectal resection, liver resection, open bowel resection, duodenopancreatectomy).<br>▪ Scheduled intraoperative volume requirement of at least 15 mL/kg body weight (Broca) gelatine solution<br>▪ Provision of voluntary consent to participate in the study, following a full explanation of the nature and purpose of the study, by signing the informed consent form approved by the Institutional Ethics Committee (IEC) prior to all evaluations.<br>Exclusion criteria:<br>▪ Patients of ASA-class > III<br>▪ Known hypersensitivity to gelatine or to any of the constituents of the solution<br>▪ Patients treated with other colloid solutions and / or blood products 24 hours prior to surgery<br>▪ Patients on hemodialysis<br>▪ Patients suffering from decompensated renal function (i.e. serum creatinine > 3.0 mg/dL)<br>▪ Patients suffering from<br>o Hypervolemia<br>o Hyperhydration

	o Severe blood coagulation disorders<br>o Hypernatremia (serum(Na^+) > 150 mmol/L)<br>o Hyperchloremia (serum(Cl^-) > 110 mmol/L)<br>o Hypercalcaemia (serum(Ca^{++} - total) > 3 mmol/L or serum (Ca^{++}-free) > 1.5 mmol/L)<br>o Metabolic alcalosis<br>o Severe heart insufficiency<br>o Severe generalized edema<br>o Intracranial haemorrhage<br>o Hyperkalaemia (serum(K^+) > 5.5 mmol/L)<br>▪ Estimated perioperative need for blood products of more than 3.5 mL/kg body weight (Broca)<br>▪ Lactation period<br>▪ Simultaneous participation in another clinical trial<br>▪ Emergencies<br>▪ Patients suffering from<br>o moderate heart or lung insufficiency<br>o moderate lung edema<br>o hypertonia<br>o eclampsia<br>who do not allow the IP regimen as required.
Investigational Test Product	The investigational test product (Gelofusine® Balanced) contains<br>Composition:<br>1000 mL solution contain<br>Gelatine polysuccinate (= modified fluid gelatine) 40.00 g<br>Sodium chloride 5.55 g<br>Sodium acetate trihydrate 3.27 g<br>Potassium chloride 0.30 g<br>Calcium chloride dihydrate 0.15 g<br>Magnesium chloride hexahydrate 0.20 g<br><br>Excipients for pH-adjustment:<br>Sodium hydroxide 40% 0 – 0.133 g<br>Hydrochloric acid 20% 0 – 0.182 g<br><br>Electrolyte concentrations<br>Sodium 151 mmol/L<br>Chloride 103 mmol/L<br>Potassium 4 mmol/L<br>Calcium 1 mmol/L<br>Magnesium 1 mmol/L<br>Bicarbonate as Acetate 24 mmol/L<br>Molecular weight, weight average : 30 000 Dalton<br>Molecular weight, number average : 23 200 Dalton

	Theoretical osmolarity : 284 mOsm/L
Investigational Reference Product	The investigational reference product (Gelafundin® 4%) contains<br>Composition:<br>1000 mL solution contain<br>Gelatine polysuccinate (= modified fluid gelatine) 40.00 g<br>Sodium chloride 7.01 g<br>Sodium hydroxide 1.36 g<br><br>Electrolyte concentrations<br>Sodium 154 mmol/L<br>Chloride 120 mmol/L<br>Molecular weight, weight average : 30 000 Dalton<br>Molecular weight, number average : 23 200 Dalton<br>Theoretical osmolarity : 274 mOsm/L
Investigational Products Administration	Method of Administration<br>The administration of the Investigational Products (colloid and crystalloid components) is performed intravenously.<br>The administration procedure starts with the treatment of the crystalloid.<br><br>Dosage<br>Administration of the investigational products will be performed intravenously, starting preoperatively before induction of anesthesia with the crystalloid.<br>The crystalloid : colloid ratio is 1 : 1 intra- and postoperatively. The administration of 1 crystalloid bag implies the administration of 1 colloid bag and vice versa. The starting infusion rate of either crystalloid or colloid is 5 mL/kg body weight (broca)/h and will be controlled by using an Infusomat.<br>The above specifications for crystalloid : colloid ratio and infusion rates are recommendations that ought to be met. However, the rates have to be adjusted according to the individual patient's needs as defined by the below triggers and targets, up to a maximum of 5 mL/kg body weight (broca)/h for the crystalloids and of 20 mL/kg body weight (broca)/h for the colloids, as considered necessary by the investigator to stabilize the hemodynamics.<br><br>The **TARGETS** for volume replacement are defined as:<br>▪ Central venous pressure between 10 and 14 mmHg (10 mmHg ≤ CVP ≤ 14 mmHg) minus PEEP after treatment with vasoactive agent<br>**and**<br>▪ Mean arterial pressure greater than 65 mmHg (MAP > 65 mmHg)

	The **TRIGGERS** for volume replacement are defined as:<br>▪ Central venous pressure is <10 mmHg (CVP <10 mmHg) minus PEEP<br>**and**<br>▪ Mean arterial pressure is <65 mmHg (MAP < 65 mmHg)<br>Postoperatively the investigational products will be given according to the patient's need.<br>**Trigger** for administration of blood products are:<br>Red blood cells (RBCs):<br>Hemoglobin (Hb) ≤ 6 g/dL<br>Hemoglobin 6 – 8 g/dL, in case of restricted compensation (e.g. coronary heart diseases, cardiac insufficiency and cerebrovascular insufficiency) and / or anemic hypoxia (e.g. tachycardia, hyopotension, ischemia and lactatacidosis)<br>Fresh frozen plasma (FFP):<br>Bleeding or aPTT > 60s or fibrinogen < 2g/dL<br>Platelets:<br>In case of bleeding or platelet count < 50.000/µL<br>Other blood products (e.g. fibrinogen, thrombocyte concentrate)<br>according to the decision of the attending physician<br>The investigational products will be applied in a blinded manner.<br>Treatment Duration<br>Treatment with the investigational products will start immediately before induction of anesthesia. It will be given intraoperatively until 12 hours after end of surgery.<br>After investigational product treatment the choice of need volume replacement is at the discretion of the attending physician.
Visit Schedule	Pre-operatively<br>▪ verification of inclusion and exclusion criteria including informed consent<br>▪ pre-operative blood sampling for laboratory measurements<br>▪ pregnancy test in urine for women of childbearing potential<br>▪ history, demographics<br>▪ Apfel-Score<br>▪ pre-operative medication / therapy

	Pre-anesthesia<br>▪ randomization<br>▪ baseline recording of hemodynamics<br>▪ blood sampling for baseline laboratory<br>▪ baseline ROTEM and baseline platelet aggregation<br>▪ set of catheters (e.g. arterial / central venous catheter)<br>▪ administration of first crystalloid bag<br>▪ drug monitoring and tolerance<br>▪ induction of anesthesia<br>▪ concomitant medication and therapy<br>▪ adverse events/ reactions<br>After induction of anesthesia / prior surgery<br>▪ administration of investigational products<br>▪ time on ventilator<br>▪ final positioning of patient in operation theatre<br>▪ set of urinary chatheter<br>▪ hemodynamics<br>▪ laboratory measurements<br>▪ drug monitoring and tolerance<br>▪ concomitant medication and therapy<br>▪ adverse events/ reactions<br>During surgery<br>▪ administration of investigational products<br>▪ surgery related data (e.g. start of intervention)<br>▪ hemodynamics<br>▪ blood sampling for laboratory measurements<br>▪ drug monitoring and tolerance<br>▪ recording of blood loss<br>▪ if applicable, blood product administration<br>▪ time on ventilation<br>▪ concomitant medication and therapy<br>▪ adverse events / reactions<br>End of surgery<br>▪ administration of investigational products<br>▪ surgery related data (e.g. end of intervention)<br>▪ hemodynamics<br>▪ blood sampling for laboratory measurements<br>▪ drug monitoring and tolerance<br>▪ recording of blood loss<br>▪ if applicable, blood product administration<br>▪ time on ventilation<br>▪ concomitant medication and therapy<br>▪ intraoperative diuresis<br>▪ adverse events / reactions<br>▪ clinical outcome<br>Admission to ICU / IMC<br>▪ administration of investigational products<br>▪ hemodynamics<br>▪ blood and urine sampling for laboratory measurements<br>▪ drug monitoring and tolerance<br>▪ if applicable, blood product administration<br>▪ if applicable, recording of secondary bleeding

	▪ if applicable, time on ventilation<br>▪ concomitant medication and therapy<br>▪ adverse events / reactions<br>6 hours after end of surgery<br>▪ administration of investigational products<br>▪ hemodynamics<br>▪ blood and urine sampling for laboratory measurements<br>▪ drug monitoring and tolerance<br>▪ if applicable, blood product administration<br>▪ if applicable, recording of secondary bleeding<br>▪ if applicable, time on ventilation<br>▪ concomitant medication and therapy<br>▪ adverse events / reactions<br>12 hours after end of surgery<br>▪ end of administration of investigational products<br>▪ hemodynamics<br>▪ blood and urine sampling for laboratory measurements<br>▪ drug monitoring and tolerance<br>▪ if applicable, blood product administration<br>▪ if applicable, recording of secondary bleeding<br>▪ if applicable, time on ventilation<br>▪ postoperative diuresis (end of surgery – 12 hours after end of surgery)<br>▪ concomitant medication and therapy<br>▪ length of stay (ICU / IMC)<br>▪ adverse events / reactions<br>▪ study termination
Duration of Study per Patient	Start of study: randomization<br>Treatment period (infusion of Investigational Products):<br>immediately before induction of anesthesia<br>until 12 hours after end of surgery<br>End of study: 12 hours after end of surgery or discharge from ICU / IMC. In case that the discharge is the latest, only its date is to be documented.
Study Schedule	Planned start: Q III 2011<br>Planned recruitment time: total sample size 1 year<br>Planned last patient out: Q III / Q IV 2012
Statistical Methods	The primary endpoints (BE and chloride) will be tested for difference between the two Gelofusine®-groups using a non-parametric statistical test (Mann-Whitney U-Test). This test is chosen with regard to small sample size and possible deviation from normal distribution.<br>All other parameters will be analysed exploratively. This analysis will also serve for testing of homogeneity of treatment groups at baseline.<br>All tests will be performed two-sided with an alpha-error α = 5%.
Randomization / Blinding	The patients will be randomised to either treatment in a 1:1 ratio.

	Randomisation will be performed in permuted blocks.<br><br>Because of the bicentre study design the population will be stratified with regard to<br><br>▪ centre
Drug Safety Monitoring Board (DSMB)	not applicable (NA)

4 INTRODUCTION

4.1 Background Information

The aim of volume replacement is to compensate a reduction in the intravascular volume e.g. during surgery and to counteract hypovolemia in order to maintain hemodynamics and vital functions. To achieve this, different plasma substitutes are available: Albumin, dextran, hydroxyethylstarches and gelatine.

Gelatine is a polypeptide produced by hydrolysis of bovine collagen. The first use of gelatine as a resuscitation infusion in hypovolemia goes back to 1915 (Hogan JJ, 1915). It was again intensively studied during the wartime in 1940 (Jacobson SD, 1944) and in 1950 (Lundsgaad-Hansen P, 1966).Today, gelatine is already used frequently during clinical routine.

There are three types of modified gelatine solutions available: cross-linked or oxypolygelatines (e.g. Gelofundiol®), urea-cross-linked (e.g. Haemaccel®) and succinylated or modified fluid gelatins (e.g. Gelafundin® 4%). Further characteristics of gelatine solutions are:

- Concentration: e.g. 3.5%, 4% or 5.5%
- Molecular weight (MW): e.g. 5000, 30000 or 50000 Dalton
- Weight-average MW: e.g. 30, 23200 or 35000 Dalton

The investigational test product Gelofusine® Balanced and the investigational reference product Gelafundin® 4% are registered products. Gelofusine Balanced is authorized in Germany since December 2011 under the brandname 'Gelafundin ISO 4%'. Both investigational products are based on the same kind of gelatine, modified fluid gelatine (MFG). The only difference is the composition of the electrolyte solution in which they are dispersed.

The investigational reference product Gelafundin® 4% is a colloidal, iso-oncotic and isotonic volume replacement fluid containing MFG in a sodium chloride solution. Its therapeutic indications are:

- prophylaxis and treatment of absolute and relative hypovolemia (e.g. following shock due to hemorrhage or trauma, perioperative blood losses,burns, sepsis)
- prophylaxis of hypotension (e. g. in connection with induction of epidural or spinal anesthesia)
- hemodilution
- extra-corporeal circulation (heart-lung machine, hemodialysis)
- increasing the leukocyte yield in leucapheresis

Besides clinical trials especially its long-standing clinical experience – it has been marketed for more than 40 years by now - revealed well-established efficacy and an excellent safety profile (Spahn D.R., 2000; Sneyd R.J. et al, 2004). The main side effects of gelatine solutions are anaphylactic reactions.

As mentioned above, the difference between the investigational reference product Gelafundin® 4% and the investigational test product Gelofusine® Balanced is the composition of the carrier solution. The carrier solution of Gelafundin® 4% consists of sodium chloride. Potassium, calcium and magnesium are additional components of the carrier solution of the new gelatine solution, Gelofusine® Balanced. Due to the fact that this composition is very close to the plasma composition it is expected that less acid base disturbances occur with the new gelatine solution. This effect has already been shown for balanced HES solutions of the 3rd generation like Tetraspan. The aim of balanced solutions is therefore to mimic the electrolyte pattern of plasma as closely

as possible in order to avoid acid-base alterations (Zander R, 2006, Wilkes 2001). The balanced concept of fluid resuscitation is a current topic in the literature (e.g. Mahler SA et al., 2010) and at congresses (e.g. Kellum JA, 2009; Trof RJ et al, 2011; Heckel K, 2011, ISICEM each). Today it is mutually accepted that acid-base alterations due to the use of unbalanced solutions should be avoided by employing a balanced volume substitute.The following table shows how this is achieved by Gelofusine® Balanced:

Table 1: Electrolyte concentrations of plasma and Gelofusine® Balanced

Electrolytes	*Plasma**	*Gelofusine® Balanced*
Sodium (mmol/L)	142.00	151.00
Potassium (mmol/L)	4.50	4.00
Calcium (mmol/L)	2.50	1.00
Magnesium (mmol/L)	1.25	1.00
Bicarbonate/Acetate (mmol/L)	24.00	24.00
Chloride (mmol/L)	103.00	103.00
Lactate (mmol/L)	1.50	--
Theoretical osmolarity (mmol/L)	291.00	284.00

*Zander R, 2006

The carrier solution of Gelofusine® Balanced reflects as far as possible the physiological anion and cation pattern as requested by Zander R, 2006.

Gelafundin® 4% has been marketed for several decades. Furthermore already other balanced gelatine solutions are on the European market which have a comparable carrier system to Gelofusine® Balanced. Hence no additional risk is expected for the patients due to the administration of the new balanced gelatine solution, Gelofusine® Balanced.

4.2 Study Rationale

In abdominal surgery intra- and postoperative volume replacement is required. Haisch et al. 2001 showed that gelatine solutions are as effective as HES solutions used in abdominal surgery patients.

It is known that infusion of large amounts of colloids in an unbalanced solution like 0.9% NaCl, leads to more-or-less discrete disturbances in acid-base balance (Wilkes, 2001). Base et al. 2011 showed that balanced HES led to a statistically significant reduction in changes of acid base balance and plasma chloride levels when compared with a non-balanced HES. Yunos et al. 2011 showed in their study that the restriction of chloride-rich fluids significantly affected electrolyte and acid-base status in intensive care e.g. the incicence of severe metabolic acidosis (standard base excess (BE) < -5 mEq/L) and decreased significantly. The BE 'removes' the respiratory element of acid-base disturbance and a metabolic acidosis can be diagnosed (Morris CG et al., 2008). Thus BE was chosen as one primary variable. Additionally chloride as another piece of the acid base status was chosen as primary variable. In summary, BE and chloride were chosen as primary variables.

Due to the fact that gelatine polysuccinate as the active substance is well-known and the electrolyte components are physiologically present in the plasma at concentrations not varying significantly from the concentrations in Gelofusine® Balanced the risk due to methods and measurements in the present study the infusion of the new gelatine solution is considered to be low.

Thus, the new gelatine solution, Gelofusine® Balanced, may be a further step into the direction of an ideal volume replacement strategy.

The aim of this prospective, randomized, controlled, double-blind, bicenter study is to demonstrate that acid-base alterations during elective abdominal surgery can be reduced by the use of a balanced gelatine solution compared to a standard gelatine solution.

4.3 Risk-Benefit-Assessment

The Investigational Products (IPs) Sterofundin® ISO, Gelafundin® 4% and Sodium Chloride 0.9% are registered in Germany as well as in various European countries. The proposed use of the Investigational Products in the present study is in line with the registered SmPCs. Gelofusine® Balanced is also registered in Germany since December 2011. The active substance MFG is well-known in clinical routine in order to perform volume replacement intra- and postoperatively. Gelatine solutions have been marketed for more than 40 years by now. All ingredients of the new gelatine solution are well-known and there are already comparable gelatine solutions with comparable carrier solutions on the market.

Clinical studies with other balanced volume replacement products have shown the positive impact of the balanced concept on acid base status.

Most of the proposed measures and parameters (laboratory, clinical) are performed/obtained routinely and thus do not represent major additional burden to the patient. Study specific measurements require additional blood samples of up to a maximum of 100 ml, which is considered a minimal risk to the patient.

In conclusion, the risk due to methods and measurements in the present study is considered to be low and a positive risk-benefit is assessed.

5 STUDY OBJECTIVES

It is the objective of the planned study to investigate the efficacy and safety of two different volume replacement regimen with gelatine solutions.

5.1 Primary Objective and Primary Variable(s)

5.1.1 Primary Objective

The primary objective of the study is to assess the intra-operative change of the base excess and chloride after treatment of a balanced volume replacement regimen (balanced gelofusine solution combined with a balanced electrolyte solution) compared with a non-balanced volume replacement regimen (non-balanced gelatine solution combined with a non-balanced electrolyte solution) in adult patients undergoing elective abdominal surgery.

5.1.2 Primary Variable

The primary variable of the study is the difference in the change of the base excess and chloride (baseline – immediately after end of surgery) after treatment between the two volume replacement regimen in adult patients undergoing abdominal surgery. (see section 10 for further details)

5.2 Secondary Objectives and Secondary Variables

Secondary objectives are safety and efficacy of the two different volume replacement regimen expressed by parameters such as arterial blood gas analysis, coagulation status, renal function, requirements of blood products, adverse events, hemodynamics, IP administration, clinical outcome, demographic data and surgery related data. (see section 10 for further details)

6 STUDY DESIGN

This is a prospective, controlled, randomized, double-blind, bicentre phase III study performed in 2 parallel groups. Comparison will be made of an Investigational Test Product (i.e. Gelofusine® Balanced) combined with a standard balanced electrolyte solution (i.e. Sterofundin® ISO) with an Investigational Reference Product (i.e. Gelafundin 4%) combined with a standard non-balanced electrolyte solution (i.e. NaCl 0.9%). In total 64 adult male and female patients will be included in the study. The aim is to study half of the patients in Aachen and the other half in Frankfurt/Main with half of the patients treated with the balanced regimen and the other half with the unbalanced regimen, each.

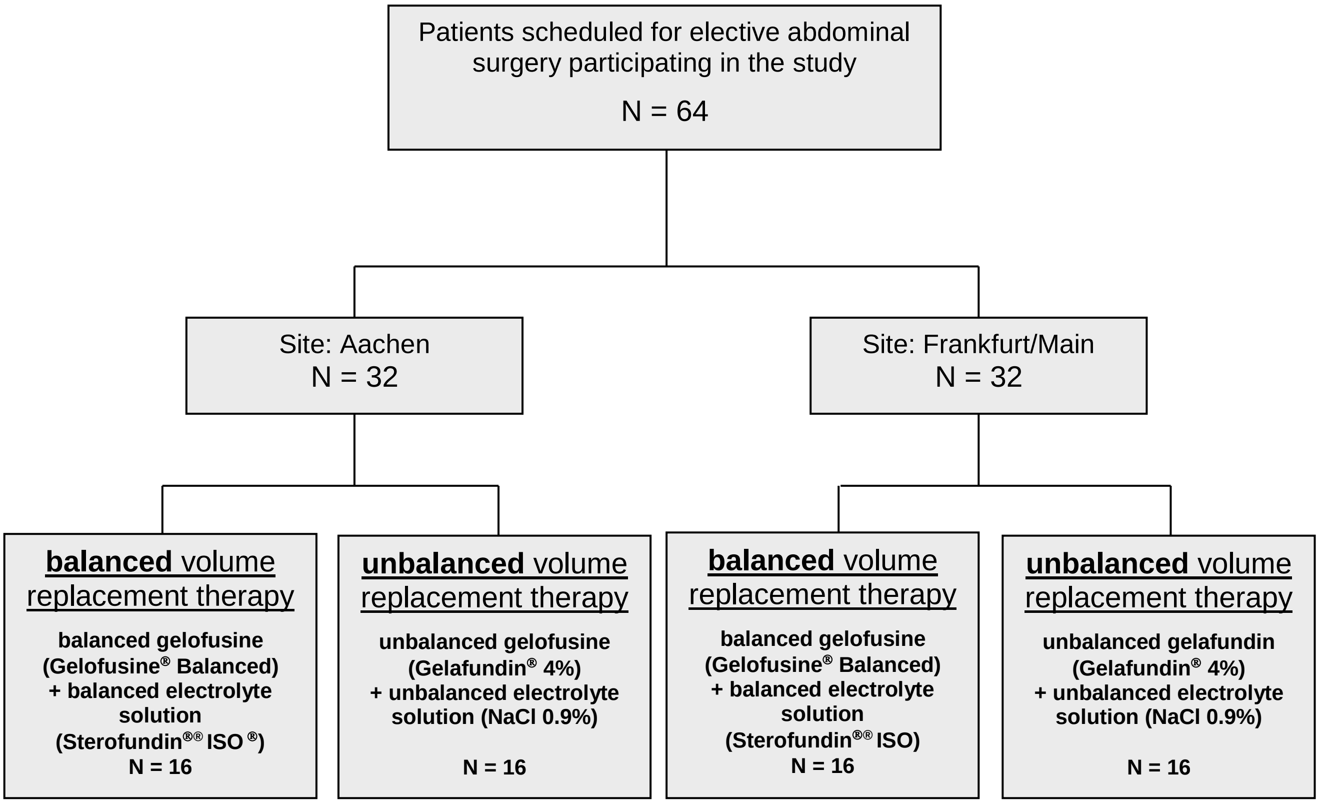

7 SELECTION AND WITHDRAWAL OF PATIENTS

7.1 Informed Consent

An informed consent, written in accordance with the origins of the Declaration of Helsinki (Appendix 1) and the applicable laws of the country has to be obtained from all patients.

The patient will sign the Informed Consent Form before s/he enters the study, i.e. before screening bloods, screening assessments or any other study-related activity. The Investigator will explain the nature, purpose and risks of the study and provide the patient with a copy of the patient information. The patient will be given sufficient time (preferably one day in advance) to consider the study's implications before deciding whether to participate.

Should there be any amendments to the Final Protocol, such that would directly affect the patient's participation in the study e.g. a change in any procedure, the Informed Consent Form must be amended to incorporate this modification and the patient's informed re-consent must be obtained.

7.2 Patient Inclusion Criteria

The following inclusion criteria apply:

- Male or female patients ≥ 18 years of age and ≤ 90 years of age.

  Women of child bearing potential must test negative on standard pregnancy test (urine dipsticks).
- Patients scheduled to undergo elective abdominal surgery (e.g. rectal resection, liver resection, open bowel resection, duodenopancreatectomy)
- Scheduled intraoperative volume requirement of at least 15 mL/kg body weigth (Broca) gelatine solution.
- Patients who are willing to give voluntary consent to participate in the study, following a full explanation of the nature and purpose of the study, by signing the informed consent form approved by the Institutional Ethics Committee (IEC) prior to all evaluations.

7.3 Patient Exclusion Criteria

Patients will not be considered for participation in the study if any of the following criteria listed below apply:

- Patients of ASA-class > III
- Patients suffering from known hypersensitivity to gelatine or to any of the constituents of the gelofusine solutions
- Patients treated with other colloid solutions and / or blood products 24 hours prior to surgery
- Patients on hemodialysis
- Patients suffering from decompensated renal function (i.e. serum creatinine > 3.0 mg/dL)
- Patients suffering from

- Hypervolemia
- Hyperhydration
- Severe blood coagulation disorders
- Hypernatriemia (serum(Na^+) > 150 mmol/L)
- Hyperchloremia (serum(Cl^-) > 110 mmol/L)
- Hypercalcaemia (serum(Ca^{++} - total) > 3 mmol/L or serum (Ca^{++}-free) > 1.5 mmol/L)
- Metabolic alcalosis
- Severe heart insufficiency
- Severe generalized edema
- Intracranial haemorrhage
- Hyperkalaemia (serum(K^+) > 5.5 mmol/L)

- Estimated perioperative need for blood products of more than 3.5 mL/kg body weight (Broca)
- Lactation period
- Simultaneous participation in another clinical trial
- Emergencies
- Patients suffering from
  - moderate heart or lung insufficiency
  - moderate lung edema
  - hypertonia
  - eclampsia

who do not allow the IP regimen as required.

7.4 Stopping and Discontinuation Criteria

When the study in an individual patient is terminated, the nature of termination must be documented (scheduled end or premature termination/discontinuation). In the event of premature termination/discontinuation, justification has to be given and it is to be recorded who took the decision to discontinue.

If the study as a whole is prematurely terminated or suspended, the concerned IEC and the regulatory authorities will be informed promptly and provided with the reasons for the termination or suspension by the sponsor.

7.4.1 Discontinuation Criteria Related to the Study

Criteria for discontinuation / termination of the entire clinical study include (not exclusively)

- Unexpected high frequency of serious adverse events/reactions during the study which does not justify a continuation of the study. (cf. Frequency as outlined in the SmPCs). Especially renal insufficiency, severe bleeding and anaphylactic reactions should be monitored. In case any of these events occurs in more than 10% of the scheduled 64 patients (i.e. 7 patients) the study is to be discontinued.

- Occurrence of suspected unexpected serious adverse reaction(s) which does/do not justify a continuation of the study.
- Patients cannot be recruited in sufficient numbers. Not sufficient is defined as recruitment of less than 2 patients per month on average.

7.4.2 Discontinuation Criteria Related to the Study Site

Discontinuation of the clinical study in an individual study site may occur because of various reasons including but not limited to

- Failure of Investigator to comply with the ICH-GCP and/or applicable regulatory requirements.
- Submission of knowingly false or incomplete information from the site to B. Braun Melsungen AG, Study Monitor, or the authorities.
- Repeated non-adherence to protocol requirements including insufficient data quality (missing data in CRFs occurring repeatedly).
- Failure of the Investigator at a site to enrol patients into the study at an acceptable rate, i.e. less than 12 patients per 6 months.
- Personnel change without appropriate information to the sponsor.

7.4.3 Discontinuation Criteria related to the Patient

The patients will be advised in the Informed Consent Forms that they have the right to withdraw from the study at any time without prejudice, and may be withdrawn at the Investigator's / Sponsor’s discretion at any time, when this is considered to be in the interest of the patient.

Withdrawal of individual patients from treatment or from the study respectively could be caused by the following reasons but not limited to

Withdrawn by the Investigator due to

- Adverse Event, Serious Adverse Event (including pregnancy)
- Protocol deviation (e.g. dosing regimen, failure to comply with protocol)
- Administration of an excluded medication
- Clinical significant abnormal laboratory value(s)

which leads to inacceptance of further remaining on treatment or in the study as considered by the investigator.

The patient requested withdrawal due to:

- An Adverse Event for which the Investigator did not consider removal from the treatment/study necessary
- Perceived insufficient therapeutic effect
- Perceived sufficient therapeutic effect
- Withdrawal of consent

In case a patient does not reappear to any scheduled visit (i. e. is lost to follow-up) reasonable effort should be made to contact this patient in order to complete

assessments and/or to evaluate reason for non-appearance (possibly implicit withdrawal of consent).

In the event that a patient withdraws consent or is withdrawn from the study, the study termination page in the CRF (including a final examination, if applicable) should be completed indicating that the study was prematurely terminated. The Investigator should record the date of the withdrawal, the person who initiated withdrawal and the reason for withdrawal.

7.5 Randomization, Blinding and Unblinding

Patients will be randomized to either treatment in a 1:1 ratio.

The list of treatment assignments considering the stratification for site will be generated by a statistician (not involved in the study data analyses) comprised of consecutive blocks with the order of assignments chosen at random (e.g.: random permuted block of size 4, 6 or 8). The randomization list will be prepared prior to the initiation of the study and will remain with the statistician. A signed copy of the randomization list will be sent to the independent pharmacy responsible for the blinding of the samples. Based on this randomization list the statistician will also issue sets of emergency envelopes for each participating site (i.e. the Principal Investiagators) for emergency unblinding. The sponsor, i.e. qualified person study supply and drug safety officer will also receive a complete set of emergency envelopes for emergency situations.

Patients who are eligible for inclusion into the study shall receive an increasing sequential patient number in accordance with the order of their inclusion in the study and according to stratum. The random numbers will be issued including center stratum (1 = Aachen or 2 = Frankfurt/Main).

Blinding will be performed by an independent pharmacy providing blinded samples. The blinded samples will be sent to the investigators who will store and manage them.

For emergency unblinding the statistician will provide sealed envelopes with the patient number printed for both the coordinating investigator and the principal investigator which will be sent to the study centers prior start of the study. When breaking the seal of an envelope the respective treatment for the individual patient is stated. The code may only be broken when this is relevant for the safety of the patient. Any premature code break (e.g. unblinding due to a Serious Adverse Event, accidental unblinding) should promptly be documented and reported to the Sponsor including explanation/justification. Emergency unblinding may be discussed with the Sponsor prior breaking the seal if possible, i.e. if this is not affecting the patient’s safety. Upon study completion all emergency envelopes will be retrieved by the study monitor.

After closure of the database and determination of the analysis populations (in a blinded data review meeting if appropriate) the study will be unblinded.

The population will only be stratified with regard to

- center

This stratification variable will be included as covariate in the primary analysis (according to CPMP/EWP/2863/99).

8 INVESTIGATIONAL PRODUCTS

Appropriate amount of the Investigational Products will be made available to the investigators organized by B. Braun Melsungen AG (Hospital Care Division). If defects in the Investigational Products are observed, the study manager or the monitor is to be informed.

8.1 Name and Description of the Investigational Product(s)

8.1.1 Qualitative and Quantitative Composition

The following table presents the composition of the Investigational Products, i.e. 1000 mL solution contain each:

	**Investigational test regime**		**Investigational reference regime**	
	Gelofusine® Balanced	***Sterofundin® ISO***	***Gelafundin® 4%***	***Sodium chloride 0.9%***
	Isotonic colloidal volume substitute	balanced electrolyte solution	colloidal volume substitute	non-balanced electrolyte solution
**Ingredients (1L)**				
Gelatine polysuccinate (=modified fluid gelatine)	40.00 g	--	40.00 g	--
Sodium chloride	5.55 g	6.80 g	7.01 g	9.0 g
Sodium acetate trihydrate	3.27 g	3.27 g	--	--
Potassium chloride	0.30 g	0.30 g	--	--
Calcium chloride dehydrate	0.15 g	0.37 g	--	--
Magnesium chloride hexahydrate	0.20 g	0.20 g	--	--
Malic acid	--	0.67 g	--	--
**Electrolyte concentrations (1L)**				
Sodium	151.0 mmol/L	140.0 mmol/L	153.0 mmol/l	154.0 mmol/L
Chloride	103.0 mmol/L	127.0 mmol/L	120.0 mmol/l	154.0 mmol/L
Potassium	4.0 mmol/L	4.0 mmol/L	--	--
Calcium	1.0 mmol/L	2.5 mmol/L	--	--
Magnesium	1.0 mmol/L	1.0 mmol/L	--	--
Acetate	24.0 mmol/L	24.0 mmol/L	--	--
Malate	--	5.0 mmol/L	--	--
**Further characteristics**				
pH	7.1 – 7.7	5.1 – 5.9	7.1 – 7.7	4.5 – 7.0
Molecular weight,	30.000 Dalton	--	30.000 Dalton	--

	Investigational test regime		Investigational reference regime	
	Gelofusine® Balanced	***Sterofundin® ISO***	***Gelafundin® 4%***	***Sodium chloride 0.9%***
	Isotonic colloidal volume substitute	balanced electrolyte solution	colloidal volume substitute	non-balanced electrolyte solution
weight average				
Molecular weight, number average	23.200 Dalton	--	23.200 Dalton	--
Theoretical osmolarity	284 mOsm/L	304 mOsml/L	274 mOsm/L	308 mOsml/L

For Gelofusine® Balanced an inhouse-specification applies while all other ingredients are in accordance to the European Pharmacopoeia (Ph. Eur.).

The Investigational Products will be manufactured and released by the Sponsor B. Braun Melsungen AG, Melsungen, Germany (Hospital Care Division) in compliance with Good Manufacturing Practice (GMP). For batch number and expiry date see certificate of analysis.

8.1.2 Pharmaceutical Form

Clear, colourless or slightly yellowish solution for infusion

8.1.3 Nature and Content of Container(s)

Polyethylene plastic bottle (Ecoflac plus®), 500 mL

Ready-to-use container for single use only. The Investigational Products should be administered immediately after connecting the container to the giving set.

8.2 Posology and Method of Administration

8.2.1 Dosage

Administration of the Investigational Products i.e. Gelofusine® Balanced + Sterofundin® ISO or Gelafundin® 4% + Sodium Chloride 0.9% in a blinded manner will be controlled individually by measuring CVP and MAP. The administration will be performed intravenously, starting preoperatively before induction of anesthesia. The following targets and triggers as in clinical routine apply:

The **TARGETS** for volume replacement are defined as:

- Central venous pressure between 10 and 14 mmHg (10 mmHg ≤ CVP ≤ 14 mmHg) minus PEEP after treatment with vasoactive agent

and

- Mean arterial pressure greater than 65 mmHg
  (MAP > 65 mmHg)

TRIGGERS for infusion of gelatines during surgery and postoperatively are:

- Central venous pressure (CVP) < 10 mm Hg minus PEEP

and

- Mean arterial pressure (MAP) < 65 mm Hg

The crystalloid : colloid ratio is 1 : 1 intra- and postoperatively. The first bottle is the crystalloid followed by the colloid and alternating thereafter. The starting infusion rate of either crystalloid or colloid is 5 mL/kg body weight (broca)/h and will be controlled by using an Infusomat.

The above specifications for crystalloid : colloid ratio and infusion rates are recommendations that ought to be met. However, the rates have to be adjusted according to the invividual patient's need as defined by the above targets and triggers, up to a maximum of 5 mL/kg body weight (broca)/h for the crystalloids and of 20 mL/kg body weight (broca)/h for the colloids, as considered necessary by the investigator to stabilize the hemodynamics.

Postoperatively the investigational products will be given according to the patient's need.

8.2.2 Method of Administration

The administration of the Investigational Products is performed intravenously.
Do not mix with other medicinal products.

8.2.3 Duration of treatment

Treatment with Investigational Product starts immediately before induction of anesthesia and reliable basal hemodynamic measurements are obtained and before skin incision.

After final positioning of the patient in the operation theatre basal hemodynamic measurements will be recorded. Prior to induction of anesthesia the Investigational Product will be given according to the above described dosage (see 8.2.1).

Treatment with Investigational Product ends 12 hours after end of surgery.

Thereafter volume replacement will be performed at the discretion of the attending physician.

8.3 Labelling

The labels were designed in accordance with GMP annex 13 and will contain the information as required by the pertinent national requirements. They must remain attached to the containers and the boxes for the duration of the clinical trial and should not be defaced.

Examples of the intended labels on the primary and secondary containers are given below. The layout and addresses may be subject to change. Variable data is indicated with “x” as placeholders.

The bottles with odd numbers contain always the crystalloid component of the respective regimen and the bottles with even numbers always the colloid component. In addition the labels of the crystalloid and colloid component will have a different colour.

Nur zur klinischen Prüfung bestimmt<br>Studien-Nr.:<br>HC-G-H-0904<br>EudraCT-Nr:<br>2010-018524-58<br><br>500 ml Infusionslösung<br>zur intravenösen Anwendung<br><br>1) Patienten-Nr. xxx<br>2) Flaschen-Nr. xx<br><br>Flaschen nur in aufsteigender Reihenfolge verwenden!<br><br>Hauptprüfarzt:	Anwendung und Dosierung<br>gemäß Studienprotokoll<br><br>Nur zu verwenden, wenn die Flasche unverletzt ist und Lösung klar und frei von sichtbaren Partikeln ist.<br>Zur einmaligen Anwendung bestimmt. Nach einem Anwendungsgang sind eventuell nicht verbrauchte Restmengen der Lösung zu verwerfen.<br>Flasche nach der Behandlung im Patientenkarton aufgebewahren.<br><br>Nicht über 25°C lagern.<br>Nicht im Kühlschrank lagern oder einfrieren.<br><br>Code-Nummer: xxxxxxx/xxxxxxx_x<br>Verwendbar bis xx/xxxx<br><br>Sponsor:<br>B. Braun Melsungen AG<br>34212 Melsungen

Nur zur klinischen Prüfung bestimmt<br>Studien-Nr.:<br>HC-G-H-0904<br>EudraCT-Nr:<br>2010-018524-58<br><br>500 ml Infusionslösung<br>zur intravenösen Anwendung<br><br>1) Patienten-Nr. xxx<br>2) Flaschen-Nr. xx<br><br>Flaschen nur in aufsteigender Reihenfolge verwenden!<br><br>Hauptprüfarzt:	Anwendung und Dosierung<br>gemäß Studienprotokoll<br><br>Nur zu verwenden, wenn die Flasche unverletzt ist und die Lösung klar und frei von sichtibaren Partikeln ist.<br>Zur einmaligen Anwendung bestimmt. Nach einem Anwendungsgang sind eventuell nicht verbrauchte Restmengen der Lösung zu verwerfen.<br>Flasche nach der Behandlung im Patientenkarton aufbewahren.<br><br>Nicht unter 10°C und nicht über 25°C lagern.<br>Nicht einfrieren.<br><br>Code-Nummer: xxxxxxx/xxxxxxx_x<br>Verwendbar bis xx/xxxx<br><br>Sponsor:<br>B. Braun Melsungen AG<br>34212 Melsungen

The labels for the secondary containers are:

Nur zur klinischen Prüfung bestimmt<br>Studien-Nr.:<br>HC-G-H-0904<br>EudraCT-Nr:<br>2010-018524-58<br><br>13 Flaschen 500 ml Infusionslösung<br>zur intravenösen Anwendung<br><br>Patienten-Nr.: xxx<br><br>Flaschen nur in aufsteigender Reihenfolge verwenden!<br><br>Hauptprüfarzt:	Anwendung und Dosierung<br>gemäß Studienprotokoll<br><br>Nur zu verwenden, wenn der Patientenkarton vor der Entnahme der ersten Flasche ungeöffnet ist, die Flaschen unverletzt sind und die Lösungen klar und frei von sichtbaren Partikeln sind.<br>Zur einmaligen Anwendung bestimmt. Nach einem Anwendungsgang sind eventuell nicht verbrauchte Restmengen der Lösung zu verwerfen.<br>Nach der Behandlung ist der Patientenkarton mit den leeren, geöffneten und ungeöffneten Flaschen aufzubewahren.<br><br>Nicht über 25°C lagern.<br>Nicht im Kühlschrank lagern oder einfrieren.<br><br>Code-Nummer: xxxxxxx/xxxxxxx_x<br>Verwendbar bis xx/xxxx<br><br>Sponsor:<br>B. Braun Melsungen AG<br>34212 Melsungen

Nur zur klinischen Prüfung bestimmt<br>Studien-Nr.:<br>HC-G-H-0904<br>EudraCT-Nr:<br>2010-018524-58<br><br>13 Flaschen 500 ml Infusionslösung<br>zur intravenösen Anwendung<br><br>Patienten-Nr.: xxx<br><br>Flaschen nur in aufsteigender Reihenfolge verwenden!<br><br>Hauptprüfarzt:	Anwendung und Dosierung<br>gemäß Studienprotokoll<br><br>Nur zu verwenden, wenn der Patientenkarton vor der Entnahme der ersten Flasche ungeöffnet ist, die Flaschen unverletzt sind und die Lösungen klar und frei von sichtbaren Partikeln sind.<br>Zur einmaligen Anwendung bestimmt. Nach einem Anwendungsgang sind eventuell nicht verbrauchte Restmengen der Lösungen zu verwerfen.<br>Nach der Behandlung ist der Patientenkarton mit den leeren, geöffneten und ungeöffneten Flaschen aufzubewahren.<br><br>Nicht unter 10°C und nicht über 25°C lagern.<br>Nicht einfrieren.<br><br>Code-Nummer: xxxxxxx/xxxxxxx_x<br>Verwendbar bis xx/xxxx<br><br>Sponsor:<br>B. Braun Melsungen AG<br>34212 Melsungen

Further principal investigator (Hauptprüfarzt) to be mentioned on the respective labels:

Study site 'Frankfurt/Main':

8.4 Packaging

The investigational products will be packed per patient with a special label on the boxes indicating for which patient of the study the samples are. The boxes contain 26 bottles per patient (i.e. 13 bottles of the colloid and crystalloid component respectively) and cover the whole study period.

The investigational products (colloid and crystalloid component) will be made available in the following type of packaging and content:

- Polythene plastic bottle (Ecoflac plus) – Colloid and crystalloid component
- 2 boxes x 13 bottles x 500 mL

8.5 Storage

The storage of the investigational products will be at a safely locked place with limited access only to authorized staff. All investigational products of the colloid component are not to be stored below 10°C and not above 25°C. The investigational products of the crystalloid component are not to be stored above 25°C and not in the fridge.

Do not freeze.

8.6 Investigational Product Accountability

In accordance with regulations, the pharmacist or other appropriate individual, who is designated by the Investigator, will keep an inventory of all clinical trial material (CTM) received. All used and unused CTM must be accounted for on an Inventory Form which will be provided to the pharmacist or other designated individual by the Site Monitor. CTM inventory forms will be examined and reconciled by the Investigator at the end of the study.

These records should include dates, quantities, batch / serial numbers, expiration dates (if applicable), and - if applicable - the unique code numbers assigned to the Investigational Product(s) and study patients. Investigators should maintain records that document adequately that the patients were provided the doses / treatments specified by the protocol.

After Investigational Product administration, i.e. 12 hours after end of surgery, all unused/opened/unopened bottles must be returned for product accountability.

A copy of the completed Inventory Form must be retained in the Investigator's study files, and a copy must be filed in the Sponsor Trial Master File.

8.7 Destruction / Retrieval of Surplus Investigational Products

Used and opened as well as surplus investigational products are to be returned to the study pharmacy after termination of the entire study. The Sponsor is responsible for the destruction of them.

9 CONCOMITANT THERAPY

Necessary concomitant medication pre-anesthesia – Patient`s standard medication

- Placement of intravenous and other catheters necessary for the infusion of the investigational products and for monitoring purposes
- Performance of hemodynamic examinations and the obtainment of blood samples for laboratory measurements (see 10)
- Medication as clinically required

Necessary concomitant medication during anesthesia and abdominal surgery:

- Noradrenalin as vasoactive treatment
- Medication as clinically required (e.g. inotropic drugs according to the medical opinion)
- Anesthesia will follow a standardized protocol of a balanced anesthesia using Sevoflurane as volatile anesthetic in combination with a peridural anesthesia where the opiod is arbitrary (left to the standard operating procedure of the study centers):
    - Thoracic peridural catheter (starting with 0.75% Naropin, followed by a mixture of Naropin 0.16% and Sufentanil 0.5 µg/mL using an infusion pump)
    - Anesthesia induction: 1 – 2 mg/kg/BW Propofol
      1 – 5 µg/kg/BW Fentanyl
      0.5 – 0.6 mg/kg/BW Rocuronium
    - Anesthesia maintenance: Sevofluran

Necessary concomitant therapy during anesthesia and abdominal surgery:

- Ventilation which is aimed at a CO_2 between 38 and 42 mmHg

Volume replacement:

- Administration of investigational products according to the **targets** and **triggers** (see 8.2)
- Administration of blood products according to the following triggers:

  Trigger for administration of red blood cells (RBCs) are:

    - Hemoglobin (Hb) ≤ 6 g/dL
    - Hemoglobin 6 – 8 g/dL, in case of restricted compensation (e.g. coronary heart diseases, cardiac insufficiency and cerebrovascular insufficiency) and / or anemic hypoxia (e.g. tahcaycardia, hyopotension, ischemia and lactatacidose)

  Trigger for administration of fresh frozen plasma are:

- Bleeding or
- aPTT > 60s or
- fibrinogen < 2g/dL

Trigger for administration of platelets are:

- Bleeding or
- platelet count < 50.000/µL

Unauthorized concomitant medication:

- Administration of other colloid or crystalloid volume replacement agents (e.g. other gelatine solutions, albumin, dextran, HES solutions, other crystalloid solutions) aside from investigational products, with exception of concentrated electrolytes for individual corrections
- Administration of sodium bicarbonate

10 DEFINITION OF THE PRIMARY AND SECONDARY VARIABLES

10.1 Primary Variable(s)

The primary variables are the course of the base excess (BE) and chloride from baseline until immediately after surgery. The difference of the measurements will represent the impact of the two different volume replacement regimes on the acid base status.

The laboratory evaluation of the BE and chloride will be done using a Radiometer.

The primary variables (BE and chloride) are used for sample size calculation (see chapter 14.5).

10.2 Secondary Variables

10.2.1 Secondary Variables – Safety

Laboratory:

The analysis of blood and urine samples will be performed by the hospital laboratories according to the corresponding methods and guidelines (see Appendix 3). The reference ranges of these parameters are given in Appendix 4.

Blood samples for laboratory parameters will be taken, if not stated otherwise, at least:

- baseline i.e. prior to induction of anesthesia
- admission to ICU / IMC
- 6 hours after end of surgery
- 12 hours after end of surgery

Additional times will be listed in the context of the respective parameter.

The results of all blood analyses performed after blood sampling will be documented as follows:

- date and time of blood sampling [dd.mm.yyyy / hh:mm].

Arterial blood gas analyses

- pondus hydrogenii (pH); not temperature corrected.
- Base excess (BE) [mmol/lL
- Bicarbonate (HCO_3) [mmol/L]
- Lactate [mmol/L]
- Sodium (Na^+) [mmol/L]
- Potassium (K^+) [mmol/L]
- Calcium (Ca^{2+}) [mmol/L]
- Chloride (Cl^-) [mmol/L]
- Partial pressure of carbon dioxid (pCO_2) [mmHg]
- Partial pressure of oxygen (pO_2) [mmHg]
- Oxygen saturation (SaO_2) [%]
- Hemoglobin (Hb) [g/dL]
- Hematocrit (Hct) [%]

Hemoglobin acts as trigger for the administration of the blood products and will be monitored intraoperatively.

Additionally to the above mentioned measurements blood samples for arterial blood gas analysis will be taken intraoperatively every 15 min including recovery room every 60 minutes and additional measurements depending on the investigators decision.

Coagulation status

- Antithrombin III (AT III) [%]
- Fibrinogen [g/dL]
- Platelet count [µ/L]
- Activated partial thromboplastin time (aPTT) [s]

Fibrinogen, activated partial thromboplastin time (PTT), and platelet count act as trigger for the administration of the blood products and will be monitored intraoperatively.

These parameters will be recorded at baseline, at admission ICU /IMC, 6 hours and 12 hours after end of surgery.

- ROTEM (CT, MCF, LI 30)
- Platelet aggregation (TRAPtest, ASPItest, ADPtest) – only site Frankfurt am Main

These parameters will be recorded at baseline, at admission ICU /IMC, 6 hours and 12 hours after end of surgery.

Renal function

Blood samples:

- Serum creatinine (SCrea) [mg/g]
- Creatinine clearance [mL/min]
- Blood urea nitrogen (BUN) [mg/dL]
- Cystatin C [mg/L]

GFR and creatinine clearance will be calculated according to the Cockroft-Gault Formula (see Chapter 14.1.1)

<u>Urin samples:</u>
Urine samples for laboratory parameters will be taken as follows:

- Amount of urine excreted (diuresis)

The urinary output will be documented intraoperatively and postoperative up to 12 hours after end of surgery:

  - Start of collection; date and time [dd.mm.yyyy / hh:mm]
  - End of collection, date and time [dd.mm.yyyy / hh:mm]
  - Total collection time in hours (will be calculated afterwards)
  - Total volume of urinary output (intra- and postoperatively) in [ml]

- N-acetyl-beta-glucosaminidase (*ß*-NAG) [units L^{-1}]
- Neutrophil gelatinase-associated lipocalin (NGAL) [ng mL^{-1}]

Urin samples for the evaluation of ß-NAG and NGAL will be taken in the same way as described above:

  - baseline
  - admission to ICU / IMC
  - 6 hours after end of surgery
  - 12 hours after end of surgery

Requirement of blood products

All blood products (e.g. RBC, FFP, platelets, etc.) applied according to the triggers during the study period will be documented separately for the time during surgery:

- Type [RBC, FFP, platelets or other including specification]
- Volume [mL]
- Start of administration [dd.mm.yyyy / hh:mm]
- End of administration [dd.mm.yyyy / hh:mm]
- Date of blood product [dd.mm.yyyy]
- Reason for administration (e.g. Hb, aPTT, bleeding, fibrinogen, platelet count)

The blood product documentation will be performed continuously during surgery.

<u>Blood loss (including secretion):</u>

The blood losses will be documented during and at end of surgery:

- Drainage blood loss; Volume [ml]
- From [dd.mm.yyyy / hh:mm]
- Through [dd.mm.yyyy / hh:mm]
- Estimated blood loss, Volume [ml]
- From [dd.mm.yyyy / hh:mm]
- Through [dd.mm.yyyy / hh:mm]

The occurrence of secondary bleeding will be documented during the whole postoperative time (i.e. starting from the end of surgery until 12 hours after end of surgery).

Adverse events

All adverse events (see chapter 11) occurring during the study period will be documented:

- Event/ symptom

- Onset of event [dd.mm.yyyy hh:mm]
- End of event [dd.mm.yyyy hh:mm]
- Intensity [mild, moderate, severe]
- Frequency [isolated, intermittent]
- Action taken [none, change in study drug dose, study drug interrupted, study drug stopped, hospitalization, concomitant therapy]
- Causal relationship to investigational product [not assessable, unlikely, possible, probable, certain]
- Serious [no, yes]
- Outcome [resolved – no sequelae; resolved with sequelae, present at final visit, death, lost to follow-up]

The adverse event documentation will be performed continuously during the study period.

10.2.2 Secondary Variables – Efficacy

Hemodynamic parameters:

- Systolic arterial pressure (SAP) [mmHg]
- Diastolic arterial pressure (DAP) [mmHg]
- Mean arterial pressure (MAP) [mmHg]
- Heart rate (HR) [beats/ min]
- Central venous pressure (CVP) [mmHg]
- Positive end-expiratory pressure (PEEP) [cmH_2O]

Please note, that a mean arterial pressure below 65 mmHg (MAP < 65 mmHg) and a central venous pressure below 10 mmHg (CVP <10 mmHg) minus PEEP are triggers for the administration of the investigational products. The crystalloid : colloid ratio is aimed intraoperatively and postoperatively 1:1. The starting infusion rate of either crystalloid or colloid is 5 ml/kg body weight (broca)/h and will be controlled by using an Infusomat.

The hemodynamic data will be measured on the contralateral site of the infusion of the investigational product.

Hemodynamic parameters will be determined:

- baseline i.e prior to induction of anesthesia
- intraoperatively every 15 min i.e. during surgery
- end of surgery i.e. wound suture
- admission to ICU / IMC
- 6 hours after end of surgery
- 12 hours after end of surgery

The documentation of the hemodynamic parameters includes the times [dd.mm.yyyy hh:mm] of assessment.

Investigational product administration:

All applied investigational product units during the study period will be documented separately for the time of administration (intra- and postoperative). The documentation of the investigational product administration will be performed continuously during the study period as follows:

- Random number
- Bottle number

- Infusion rate [mL/kg]
- Start of administration [dd.mm.yyyy / hh:mm] per infusion rate
- End of administration [dd.mm.yyyy / hh:mm] per infusion rate
- Reason for administration

The administration will be in compliance with the specified algorithm in chapter 8.2.

Drug monitoring and tolerance:

The drug tolerance will be monitored during the study period:

- Have there been any technical/ surgical problems?
  [yes, no] [specification in case of 'yes'] [dd.mm.yyyyy]
- Any allergic reactions (e.g. pruritus, flush)?
  [yes, no] [specification in case of 'yes'] [dd.mm.yyyyy]
- Any circulatory or respiratory problems?
  [yes, no] [specification in case of 'yes'] [dd.mm.yyyyy]
- Any other clinical symptoms or adverse events?
  [yes, no] [specification in case of 'yes'] [dd.mm.yyyyy]

The documentation of drug monitoring and tolerance will be performed during investigational product administration.

Concomitant medication and therapy:

All medication given during the study period including pre-anesthetic medication will be documented as follows:

- Drug, active substance
- Dose, unit and given amount (only for active substance / drug)
- Route of administration [intravenous (iv), intramuscular (im), oral (po), other]
- Start of administration [dd.mm.yyyy / hh:mm]
- End of administration [dd.mm.yyyy / hh:mm]
- Indication

All therapies performed during the study period will be documented as follows:

- Kind of therapy
- Start of therapy [dd.mm.yyyy]
- End of therapy [dd.mm.yyyy]
- Indication

Concomitant medication and therapy will be documented continuously during the study period for the time prior to anesthesia until 12 hours after surgery.

10.3 Other variables

<u>Demographic data</u>

- Age [years]
- Height [cm]
- Weight [kg]
- Sex
- ASA-classification
- Apfel-Score
- Concomitant disease

- Diagnosis

Measurements:
- ◦ pe-operatively / prior to anesthesia

Surgery related data
- Kind of intervention / precise classification of surgical intervention
- Date of intervention [dd.mm.yyyy]
- Start and end of anesthetics application [hh:mm]
- Start and end of intubation [hh:mm]
- Time of incision [hh:mm]
- Time of wound suture [hh:mm]

Measurements:
- ◦ during / after surgery

Clinical Outcome
- Time on ventilator
- Ventilator settings (i.e. frequency, minute volume, ventilation pressure)

Measurements:
- ◦ measured from intubation in hours or in case of setting changes
- ◦ reintubation will be documented
- ◦ requirements of postoperative ventilatory support will be documented

- Secondary bleeding

Measurements:
- ◦ after end of surgery until 12 hours after end of surgery

- Length of stay on ICU / IMC

Measurements:
- ◦ admission to ICU / IMC
- ◦ discharge from the ICU / IMC

10.4 Source Documents

For definitions of source documents and data see section 15.

The following table represents the source documents for the data to be entered in the CRF:

Variable	Source document
Informed consent form	Informed consent form
Inclusion and exclusion criteria	Patient chart
Patient's data (demographics: sex, date of birth, weight, height, diagnosis, concomitant diseases, Apfel-Score)	Patient chart
Indication, operative measures, surgery related data	Anesthesia chart
ICU / IMC stay	Patient chart
Investigational product administration	Intraoperative and postoperative charts
Blood product administration	Patient chart
Blood loss	Patient chart

Variable	Source document
Drug monitoring and tolerance	Patient chart
Concomitant medication and therapy	Intraoperative and postoperative charts
Hemodynamics	Intraoperative and postoperative charts
Laboratory measures	Patient chart / Laboratory, device print-out
Adverse events	Intraoperative and postoperative charts, CRF, SAE-form
Clinical outcome	Patient chart
Final assessment	CRF
Study termination	CRF, patient chart

11 ASSESSING AND REPORTING OF ADVERSE EVENTS

Throughout the course of the clinical trial particular attention is paid to Adverse Events (AE) and Adverse Drug Reactions (AR).

11.1 Definitions

11.1.1 Adverse Events

An Adverse Event (AE) is any untoward medical occurrence in a patient or clinical investigation subject administered a pharmaceutical product and which does not necessarily have a causal relationship with this treatment. An AE can therefore be any unfavourable and unintended sign (including an abnormal laboratory finding), symptom, or disease temporally associated with the use of an Investigational Product, whether or not related to the investigational product or study treatment/procedures.

11.1.2 Adverse Reaction

Adverse Reactions (AR) are all untoward and unintended responses to an Investigational Product related to any application / dose administered. All adverse events judged by either the reporting investigator or the sponsor as having a reasonable causal relationship to a medicinal product qualify as AR. The expression reasonable causal relationship means to convey in general that there is evidence or argument to suggest a causal relationship.

Regarding marketed Investigational Products: a response to a product which is noxious and unintended and which occurs at applications normally used in man for prophylaxis, diagnosis, or therapy of diseases or for modification of physiological function.

11.1.3 Unexpected Adverse Reactions

An AR, the nature or severity of which is not consistent with the applicable product information (= reference document, e.g. Investigator's Brochure (IB) for an unauthorised Investigational Product or SmPC for an authorised product).

When the outcome of the AR is not consistent with the applicable product information/reference document this AR should be considered as unexpected.

The reference document is identified in section 17.8. Refer also to section 11.4.

11.1.4 Serious Adverse Event (SAE) or Serious Adverse Reaction (SAR)

Any untoward medical occurrence or effect that at any dose

- Results in death
- Is life threatening
- Requires hospitalisation or prolongation of existing inpatients' hospitalisation
- Results in persistent or significant disability or incapacity
- Is a congenital anormaly or birth defect

More than one of the above criteria can be applicable to the one event.

NOTE:

Death: is the outcome of an AE. The event to be reported comprehensively is the medical condition leading to death, e.g. underlying disease, accident.

Life-threatening: in the definition of a SAE or SAR refers to an event in which the patient was at risk of death at the time of the event; it does not refer to an event which hypothetically might have caused death if it was more severe.

Hospitalisation is defined as inpatient care of more than one calendar day (overnight admission). Admission for ambulant diagnostic procedures, overnight survey visits or ambulant visits to an emergency ward, e.g. during weekends are not considered 'hospitalisation' in the sense of the criteria for SAE / SAR, unless any of the other criteria for serious is met.

Medical judgement should be exercised in deciding whether an AE / AR is serious in other situations. Important: Aes / Ars that are not immediately life-threatening or do not result in death or hospitalisation but may jeopardise the patient or may require intervention to prevent one of the other outcomes listed in the definition above, should also be considered serious.

11.1.5 Adverse Event Intensity

**Intensity**	**Definition**
Mild	Patient is aware of signs and symptoms but they are easily tolerated
Moderate	Signs / symptoms cause sufficient discomfort to interfere with usual activities
Severe	Patient is incapable to work or perform usual activities

Cave: The term "severe" is often used to describe the intensity (severity) of a specific event. This is not the same as 'serious', which is based on patient/event outcome or action criteria (see definition 11.1.4)!

11.1.6 Adverse Event Causality

Causality code	Definition
Not assessable	A report suggesting an AE, which cannot be judged because information is insufficient or contradictory, and which cannot be supplemented or verified.
Unlikely	A clinical event, including laboratory test abnormality, with a temporal relationship, which makes a causal relationship improbable, and in which other drugs / treatments, chemicals or underlying disease(s) provide plausible explanations.
Possible	A clinical event, including laboratory test abnormality, with a reasonable temporal relationship to administration of the drug / treatment, but which also could be explained by concomitant diseases or other drugs / treatments or chemicals.
Probable	A clinical event, including laboratory test abnormality, with a reasonable temporal relationship to administration of the drug / treatment, unlikely to be attributable to concomitant disease(s) or other drugs / treatments or chemicals, and which follows a clinically reasonable response on withdrawal (dechallenge). Rechallenge information is not required to fulfil this definition.
Certain	A clinical event, including laboratory test abnormality, occurring in a plausible time relationship to study treatment and which cannot be explained by concomitant disease(s), other drugs / treatments or chemicals. The response to withdrawal of the treatment (dechallenge) should be clinically plausible. The event must be unambiguously either pharmacologically or as phenomenon, using in satisfactory rechallenge procedures if necessary.

11.2 Recording and Reporting Adverse Events and Adverse Reactions

11.2.1 Recording

The Investigator must record in detail all Aes (signs and symptoms) which are either volunteered by patients or observed during or following the course of Investigational Products administration and during the course of the study on the appropriate CRF page.

Included in the description should be

- the nature of the sign or symptom;
- the date of onset; date of resolution (duration);
- the severity / intensity (for definition see section 11.1.5);
- the investigator’s judgement on possible relationship to study treatment or other therapy (for definition see section 11.1.6);
- the action taken (if any), and
- the outcome.

11.2.2 Reporting of Serious Adverse Events and Unexpected Adverse Reactions

All SAEs, whether or not deemed Investigational Product-related or expected must be reported to the Sponsor by telephone within 24 hours (one working day) of the Investigator becoming first knowledge:

Contact Name: see "Responbilities and Addresses (section 1)

A written report must follow within five working days and is to include a full description of the event and sequelae, in the format detailed by the Serious Adverse Event reporting form provided by the Sponsor (see Appendix 3).

The Sponsor will notify the competent authorities, IECs and all investigators concerned of suspected SARs which are unexpected (SUSARs) in line with pertinent legal requirements.

While reporting adverse events, all pertinent data protection legislation must be adhered to.

Exempted from reporting within 24 hours are all SAEs which occurred before treatment with the gelatine component of the investigational regimen (bottle number 2), i.e. from induction of anesthesia until start of second bottle of investigational product treatment.

11.3 Adverse Event follow-up procedures

Adverse Events will be followed up throughout the course of the clinical trial.

11.4 Potential Risks and Potential Adverse Events

The proposed treatment is in line with normal procedures in abdominal surgery . However, the active substance of Gelofusine® Balanced, gelatine polysuccinate (= modified fluid gelatine), is a well-known active substance in the field of volume replacement over several decades. The difference between the well known product Gelafundin® 4% and the new gelatine solution Gelofusine® Balanced is the composition of the electrolyte carrier solution. This, in turn, represents a composition of electrolytes close to the composition of the blood plasma and is comparable with the carrier solution of other balanced gelofusine solutions like Isoplex® 4% or Geloplasma® and HES solutions like Tetraspan®. For the new gelatine solution Gelofusine® Balanced the same adverse drug reactions as for Gelafundin® 4% will apply.

According to the SmPCs for Gelafundin® 4%, for Gelafundin ISO 40 mg/ml, for Sterofundin® ISO and for Sodium chloride 0.9% the following adverse drug reactions are described:

Gelafundin ISO 40 mg/ml / Gelafundin® 4%:

Although anaphylactoid reactions are rare these are the most common side effects of gelofusine solutions.

<u>Immune system disorders</u>

Rare: Anaphylactoid reactions (all grades)

After the administration of gelatine solution infusions, just as of any colloidal volume substitutes, anaphylactoid reactions of varying degrees of severity may occur. These reactions manifest as cutaneous eruptions and may proceed over sudden flushing of

the face and neck to a drop in blood pressure, shock, cardiac and respiratory arrest. Severe anaphylactoid reactions (grade III or IV) are very rare (incidence < 1 : 10 000). Patients receiving gelatine solutions must be continuously observed for the occurrence of anaphylactoid reactions.

Gastrointestinal disorders

Uncommon: Transient mild nausea or abdominal pain

General disorders

Uncommon: Transient mild rise of body temperature

The following general guidelines for the prophylaxis of adverse reactions apply:

- Adequate information of physicians and nursing staff about the type and severity of possible adverse reactions that may be encountered after the administration of a colloidal volume substitute.
- Careful observation of the patient during infusion, especially while the first 20 – 30 ml of the solution are being infused.
- Immediate availability of all equipment and medication for cardio-pulmonary resuscitation.
- Immediate stop of infusion as soon as there are any signs of adverse reactions.

Emergency treatment of anaphylactoid reactions follows established schedules, depending on the severity of the reaction. It cannot be predicted by any test procedure which patients are likely to experience anaphylactoid reactions, nor is it possible to foresee the course and severity of any such reaction.

Anaphylactoid reactions caused by gelatin solutions may either be histamine mediated or histamine independent. Histamine release can be prevented by the use of a combination of H1- and H2 receptor blockers.

Prophylactic administration of corticosteroids has not proven effective.

Adverse reactions may occur in conscious and anesthetized patients. In the acute phase of volume deficiency shocks so far no anaphylactoid reaction has ever been reported.

Sterofundin® ISO:

Overdose or too fast administration may lead to water and electrolyte (including acetate, malate) overload with respective signs and symptoms as described in SmPC section “Overdose”.

Gastrointestinal disorders:

Rare: Although oral magnesium salts stimulate peristalsis, paralytic ileus has beenrarely reported after intravenous infusion of magnesium sulphate.

General disorders and administration site conditions:

Adverse reactions may be associated with the technique of administration including febrile response, infection at the site of injection, local pain or reaction, vein irritation, venous thrombosis or phlebitis extending from the site of injection and extravasation.

Immune system disorders

Not known: Hypersensitivity reactions characterized by urticaria have been occasionally described after the intravenous administration of magnesium salts.

Sodium Chloride 0.9%:

Overdose may result in hypernatremia, hyperchloremia, overhydration, hyperosmolarity of the serum, and metabolic acidosis.

Administration of larger amounts may lead to hypernatremia and hyperchloremia (see also SmPC section “Overdose”).

11.5 Pregnancies

Not applicable, because the study is performed in the operation room and the ICU / IMC.

12 STUDY SCHEDULE

12.1 Procedures at Each Visit

Pre-operatively:

- Verification of inclusion and exclusion criteria, including informed consent
- Blood samples for laboratory evaluation of electrolyte and coagulation status
- Urine sample in women of child bearing potential for pregnancy testing
- Demographic data, history
- Apfel-Score
- Pre-operative medication / therapy as medically indicated and standardized (including pre-operative fasting time)

Pre-anesthesia:

- Randomization (i.e. supply of blinded study samples)
- Set of catheters (e.g. arterial / central venous catheter)
- Baseline laboratory and hemodynamics
- Blood sampling for baseline laboratory evaluation
- Baseline ROTEM and baseline platelet aggregation
- Administration of first bottle of investigational products, i.e. crystalloid component
- Drug monitoring and tolerance
- Induction of anesthesia
- Concomitant medication and therapy (including concomitant medication and therapy prior induction of anesthesia; including blood products acc. to trigger)
- Adverse events/ reactions

After induction of anesthesia/prior surgery :

- Administration of investigational products
- Time on ventilator
- Final positioning of patient in operating theatre
- Set of urinary catheter for urine sampling
- Hemodynamics
- Blood sampling for laboratory evaluation
- Drug monitoring and tolerance
- Concomitant medication and therapy (including concomitant medication and therapy prior induction of anesthesia; including blood products acc. to trigger)
- Adverse events / reactions

During surgery :

- Administration of investigational products for volume replacement
- Surgery related data
- Intraoperative hemodynamics (every 15 min; CVP minus PEEP and MAP serve as trigger for IP-administration)
- Blood sampling for laboratory evaluation (blood gas analysis every 15 minutes, recovery room hourly)
- Drug monitoring and tolerance
- Recording of blood loss (drainage blood loss and estimated blood loss)
- if applicable, blood product administration
- Time on ventilation
- Concomitant medication and therapy
- Adverse events / reactions

End of surgery:

- Administration of investigational products for volume replacement according to algorithm
- Surgery related data
- Hemodynamics
- Blood sampling for laboratory evaluation
- Drug monitoring and tolerance
- Recording of blood loss (drainage blood loss and estimated blood loss)
- if applicable, blood product administration
- Time on ventilation
- Concomitant medication and therapy

- Intraoperative diuresis: cumulated duiresis from start of anesthesia until end of surgery
- Adverse events / reactions

Admission to ICU / IMC:

- Administration of investigational products for volume replacement according to algorithm
- Hemodynamics
- Blood and urine sampling for laboratory evaluation
- Drug monitoring and tolerance
- Administration of blood products, if applicable
- Recording of secondary bleeding, if applicable
- Time on ventilation, if applicable
- Concomitant medication and therapy
- Adverse events / reactions
- Clinical outcome

6 hours after end of surgery:

- Administration of investigational products for volume replacement according to algorithm
- Hemodynamics
- Blood and urine sampling for laboratory evaluation
- Drug monitoring and tolerance
- Administration of blood products, if applicable
- Recording of secondary bleeding, if applicable
- Time on ventilation
- Concomitant medication and therapy
- Adverse events / reactions

12 hours after end of surgery:

- End of administration of investigational products for volume replacement according to algorithm
- Hemodynamics
- Blood and urine sampling for laboratory evaluation
- Drug monitoring and tolerance
- Administration of blood products, if applicable
- Recording of secondary bleeding, if applicable
- Postoperative diuresis (admission ICU / IMC – 12 hours after end of surgery)

- Time on ventilation
- Concomitant medication and therapy
- Length of stay (ICU / IMC)
- Adverse events / reactions
- Study termination

12.2 Tabular Overview

TIME / PROCEDURE	Pre-operative	***Randomization***	Prior to induction of anesthesia	After induction of anesthesia	During surgery	End of surgery	Admission to ICU / IMC	6 hours after end of surgery	12 hours after end of surgery / ICU/IMC discharge (which occurs latest)
Inclusion / exclusion criteria	x								
Written informed consent	x								
Pregnancy test (woman)	x								
History, demographics, Apfel-Score	x								
pre-operative medication	x								
Randomization		x							
Surgery related data					x	x			
Time on ventilator				x	x	x	x	x	x
Ventilator settings				x	x	x	x	x	x
Set of catheters and final positioning of patient			x	x					
Infusion of the investigational test or reference products			continuously[h]						
Hemodynamics			x[a]	continuously[e]					
Drug monitoring and tolerance			continuously						
Drainage blood losses and estimated blood loss					x	x			
Set of urinary catheter			x						
Recording of							continuously		

TIME / PROCEDURE	Pre-operative	*Randomization*	Prior to induction of anesthesia	After induction of anesthesia	During surgery	End of surgery	Admission to ICU / IMC	6 hours after end of surgery	12 hours after end of surgery / ICU/IMC discharge (which occurs latest)
secondary bleeding									
Laboratory (blood and / or urine sampling)			x[a]	x[b]	x[b]	x[b]	x	x	x[g]
Blood gas analysis			x	x[f]	x[f]	x[f]	x	x	x[g]
Coagulation			x				x	x	x[g]
ROTEM			x				x	x	x[g]
Platelet aggregation (only site Frankfurt / Main)			x				x	x	x[g]
Renal function			x				x		x[g]
(Serious) adverse events / reactions			continuously						
Concomitant medication incl. blood products			continuously						
Diuresis						x[c]			x[d]
Length of ICU / IMC stay									x[i]
Study termination									x[d]

a baseline

b Hb, aPTT, fibrinogen and PC serve as trigger for blood product-administration;
blood gas analysis intraoperatively every 15 min including recovery room every hour

c cumulated diuresis from start of anesthesia until end of surgery

d cumulated diuresis from end of surgery until 12 hours after end of surgery or ICU/IMC discharge, which occurs latest

e intraoperative every 15 min; CVP minus PEEP and MAP serve as trigger for IP-administration

f intraoperative every 15 min, recovery room hourly

g 12 hours after end of surgery

h until 12 hours after end of surgery

I discharge from ICU / IMC

12.3 Assessment of Compliance

not applicable

12.4 Precautionary Measures

The following measures are listed in the repective SmPCs:

Gelafundin ISO 40 mg/ml / Gelafundin® 4%:

Gelatine solutions should be administered with caution to patients with a history of allergic diseases, e.g. asthma.

Gelatine preparations for volume replacement may rarely cause anaphylactoid reactions of varying degrees of severity. In order to detect the occurrence of an anaphylactoid reaction as early as possible, the first 20 – 30 ml should be infused slowly and under careful observation of the patient. Details of symptoms of anaphylactoid reactions and emergency measures, see section 11.4.

Attention must also be paid to the dilution of plasma proteins (e.g. albumin and coagulation factors), which must be adequately substituted if necessary.

Gelatine solutions should only be administered with caution to:

- elderly patients,
- patients at risk of circulatory overload e.g. patients with congestive heart failure, right or left ventricular insufficiency, hypertension, pulmonary oedema or renal insufficiency with oligo- or anuria,
- patients suffering from coagulation disorders, chronic liver disease, lung edema, intracranial hemorrhage.

In such cases gelatine solutions should only be given under careful monitoring of the patient's hemodynamic situation.

There is no sufficient experience with the use of gelatine solutions in children. Therefore, gelatine solutions should be used in children only after careful benefit-risk assessment, and with careful monitoring.

In states of dehydration the fluid deficit must be corrected first. Electrolytes should be substituted as required.

Checks of serum electrolyte concentrations and water balance are necessary, in particular in patients with coagulation disorders, chronic liver disease, hypernatremia, hypokalemia, dehydration, or impairment of renal function.

Special attention should be paid to the appearance of symptoms of hypocalcemia (e.g. signs of tetany, paresthesia); then specific corrective measures should be taken.

During compensation of severe blood losses by infusions of large amounts of gelatine solutions , the hematocrit must be monitored under any circumstances. The hematocrit should not decrease below the critical values of 25% in general and 30% in ICU patients.

Likewise in those situations the dilution effect on coagulation factors should be observed, especially in patients with existing disorders of hemostasis.

Because the product does not substitute lost plasma protein, it is advisable to check the plasma protein concentrations.

Gelatine solutions must not be infused through the same infusion line together with blood or blood products (packed cells, plasma and plasma fractions).

Laboratory blood tests (blood group or irregular antigens) are possible after gelatine infusions. Nevertheless it is recommended to draw blood samples before the infusion of gelatin solutions in order to avoid hampered interpretation of results.

Gelatine solutions may have an influence on the following clinical-chemical tests, leading to falsely high values:

- erythrocyte sedimentation rate,
- specific gravity of urine,
- unspecific protein assays, e.g. the biuret method.

Sterofundin ISO:

High volume infusion must only be used under specific monitoring in patients with mild to moderate cardiac or pulmonary failure.

Solutions containing sodium chloride should be administered with caution to patients with

- mild to moderate cardiac insufficiency, peripheral or pulmonary oedema or extracellular hyperhydration
- hypernatremia, hyperchloremia, hypertonic dehydration, hypertension, impaired renal function, present or imminent eclampsia, aldosteronism or other conditions or treatment (e.g. corticoids/steroids) associated with sodium retention

Solutions containing potassium salts should be administered with caution to patients with cardiac disease, conditions predisposing to hyperkalemia such as renal or adrenocortical insufficiency, acute dehydration, or extensive tissue destruction as occurs with severe burns.

Because of the presence of calcium

- Care should be taken to prevent extravasation during intravenous infusion.
- The solution should be given cautiously to patients with impaired renal function or diseases associated with elevated vitamin D concentrations such as sarcoidosis.

In case of concomitant blood transfusion, the solution must not be administered via the same infusion set.

Solutions containing metabolisable anions should be administered cautiously to patients with respiratory impairment.Monitoring of the serum electrolytes, fluid balance, and pH is necessary.

During long-term parenteral treatment, a convenient nutritive supply must be given to the patient.

Sodium Chloride 0.9%:

Sodium Chloride 0.9% should only be administered with caution in cases of

- hypokalemia
- hypernatremia
- hyperchloremia

- disorders where restriction of sodium intake is indicated, such as cardiac insufficiency, generalized oedema, pulmonary oedema, hypertension, eclampsia, severe renal insufficiency.

Clinical monitoring should include checks of the serum ionogram, the water balance, and theacid-base status.

High infusion rates should be avoided in cases of hypertonic dehydration because of possible increases of plasma osmolarity and plasma sodium concentration.

Each patient will be checked if he/she can be treated according to the protocol or not. Patients at risk should be treated with caution and be excluded if the IP regimen can not be applied as required.

13 STUDY AND TREATMENT DURATION

13.1 Duration per patient

Start of study: with randomization prior to anesthesia

Treatment period (administration of Investigational Products):

Start of administration: immediately before start of anesthesia

Duration of administration: intraoperatively and postoperatively until 12 hours after end of surgery

End of study: 12 hours after end of surgery or discharge from ICU / IMC. In case that the discharge is the latest, only its date is to be documented.

13.2 Duration of whole study

Planned study start: Q III 2011

Planned recruitment time: 1 year

Planned last patient out: Q III / Q IV 2012

14 STATISTICS

14.1 Statistical methods

This section gives an overview of the statistics planned for the study. All programming of tables, figures, listings and statistical analyses will be performed using the statistical software packages as SPSS® or SAS®. The planned statistics will be performed in accordance with the principles outlined by the guideline ICH E9.

A Statistical Analysis Plan (SAP) will be finalized before close of database. It includes all protocol amendments. In case a protocol amendment is passed after finalization of the SAP an amendment to the SAP is to be composed.

All target criteria (primary and secondary variables) will first be examined by exploratory data analysis and descriptively evaluated (Wernecke 1995). In this setting, the evaluation of structural homogeneity of the treatment groups will be performed for

the purpose of quality assurance. The primary endpoint (difference in acid base change between balanced and non-balanced volume replacement with gelatine solutions) will be evaluated with a non-parametric statistical test (Mann-Whitney U-test, Hartung and Elpelt, 1993) taking into consideration small sample sizes and possible deviation from normal distribution. The stratification variable 'site' will be included in the primary analysis as covariates (in a nonparametric analysis of covariance cf. Bathke, Brunner 2003).

Secondary target variables will also be evaluated with nonparametric tests according to their scaling. Whereby, in case of a small random sample size or an unbalanced condition, exact tests will be used (Mehta, Patel, 1998).

The following parameters will be calculated by the given formula in the course of the statistical analysis:

- Anion gap [mmol/L] using
  Anion gap = Sodium + Potassium – Bicarbonate – Chloride
- Strong ion deficit (SID) [mmol/L] using
  Strong ion difference = Sodium + Potassium – Chloride – Lactate
- Creatinine clearance / GFR using Cockcroft-Gault-Formula
  Creatinine clearance
  = ((140 – age) x weight) / (72 x Serum creatinine) for male patients and
  Creatinine clearance
  = ((140 – age) x weight) / (72 x Serum creatinine) x 0.85
  for female patients
- Ratio of applied blood product volume and estimated blood volume of the patient
- Ratio of time on ventilation and time of intervention (i.e. from skin incision until skin suture)

14.2 Interim Analysis

The confirmation respective recalculation of sample size for the first primary variables after inclusion of 40 patients will only be performed by estimating the pooled variance (without unblinding). (Refer to section 14.5)

14.3 Level of significance and power

All tests will be conducted with an error of 1st kind α = 5%, two-sided.

Tests of secondary variables will be carried out in the area of exploratory data analysis. Therefore, corresponding p-values are to be regarded as exploratory ones and no adjustments for multiple testing will be made.

Sample size calculations are to be performed with a power of 80%.

14.4 Statistical hypotheses

The primary objective is the verification of a different change in acid base status (ABS) using a balanced gelatine solution combined with a balanced electrolyte solution versus a non-balanced gelatine solution combined with a non-balanced electrolyte solution, so that the following hypotheses need to be tested:

H_0 (null hypothesis):
ABS [balanced] = ABS [non-balanced]

H_A (alternative hypthesis, two-sided):
ABS [balanced] $\neq$ ABS [non-balanced]

14.5 Sample Size

The primary variables, i.e. base excess (BE) and chloride, are used to calculate the sample size. The data of a study investigating a balanced HES (Wilkes, 2001) served as the basis for the sample size calculation. Due to the fact that it was a comparison of two HES-solutions the results are only an orientation. Therefore these data were adapted. Due to the fact that in this study the reference gelatine solution is already slightly balanced the mean effect regarding chloride were reduced.

Sample size calculation using nQuery Advisor 7.0 resulted in a sample size of 56 patients in total. It was based on the following assumptions:
11 patients per group for chloride, supposing an

- effect size (difference of means/common standard deviation) = 1,711 with difference in means = 6.500.0 and common standard deviation = 3.800,
- error of 1st kind $\alpha = 2.5\%$ (two-sided),
- power = 80% and
- the nonparametric Wilcoxon (Mann-Whitney) test.

28 patients per group for BE, supposing an

- effect size (difference of means/common standard deviation) = -0.910 with difference in means = -3.5 and common standard deviation = 3.847,
- error of 1st kind $\alpha = 2.5\%$ (two-sided),
- power = 80% and

the nonparametric Wilcoxon (Mann-Whitney) test.

In order to account for both parametes and taking a drop out rate of 10% into account, a sample size of 32 patients per group (i.e. 64 patients in total) will be needed.

However, at present no reliable information is available on the acid base status with the new gelatine solution. Thus, an internal pilot phase including 40 patients (including drop-outs) will be performed in order to re-calculate total sample size. For re-calculation the pooled variance will be assessed on the grounds of clinical relevant difference without unblinding. Type I error for both phases of the study will strictly be maintained. (Kieser and Friede, 2000 and 2003).

After sample size adjustment the responsible ethic committees will be asked for approval of study continuation or for termination (e.g. due to futility).

Withdrawn patients will not be replaced because they are considered in the drop out rate.

14.6 Data handling

All data will be listed. Whenever applicable all tables, figures and listings will identify patients using the patient identification (ID) and time of evaluation. For data collected prior to randomization, patients will appear in the listings with the treatment to which they were subsequently allocated.

The actual population presented in a table/figure/listing will be mentioned in the headings.

Tables and listings will be produced in accordance with the principles outlined by the ICH E3 guideline.

Handling missing data and outliers

Every effort will be made to collect all data points in the study. The amount of missing data will be minimized by appropriate management of the randomized, prospective, trial, proper screening of subjects, and training of participating investigators and other authorized staff (e.g. nurses), monitors and study co-ordinator. Since all patients who are randomized will be included in the primary analysis, in those instances where data are missing, missing values will not be imputed.

Missing data in tables and listings will be handled as missing data by inserting the symbol '.'.

Outliers may be identified using stem-leaf plots and frequency distributions, Scatter and box plots may be also generated for outlier identification. For normally distributed data, values more than three standard deviations away from the mean will be considered outliers. Transformation of the data to mitigate the influence of outliers may be considered. If outliers remain, additional analyses excluding these values will be done and discussed in the report.

14.7 Criteria for the termination of the study

not applicable, because no interim analysis is planned

14.8 Patient selection for analyses

An Intent-To-Treat (ITT) analysis and Full Analysis Set (FAS) respectively is planned as primary.

Additionally, an Per-Protocol (PP) or Valid Case Analysis Set (VCAS) respectively will be performed i. e. all patients where none of the following events occurred:

- Stop of treatment due to adverse reaction
- severe violation of study protocol

The results of both analyses will be compared and possible differences have to be discussed accordingly.

The definitions for any population groups chosen for analysis (e.g. all patients randomized, intent-to-treat, per-protocol, completers) will be provided in the SAP. Decision on analysis of the respective study population will be performed prior unblinding.

15 SOURCE DATA AND SOURCE DOCUMENTS

15.1 Definitions

15.1.1 Source Documents

Source documents are defined as original documents, data and records (e.g. hospital records, clinical and office charts, laboratory notes, memoranda, patient diaries or evaluation check lists, pharmacy dispensing records, recorded data from automated

instruments, copies or manuscripts certified after verification as being accurate copies, microfiches, photographic negatives, microfilm or magnetic media, x-rays, patient files, records kept at pharmacy, at the laboratories and at medico technical departments involved in clinical study).

Refer to section 10.4 which documents serve as source documents for the present study.

15.1.2 Source Data

Source data are defined as all information in original records and certified copies of original records of clinical findings, observations or other activities in a clinical study necessary for the reconstruction and evaluation of the study. Source data are contained in source documents (original records or certified copies).

15.1.3 Direct Access

Direct access is defined as the permission to examine, analyse, verify and reproduce any records and reports that are important to evaluation of a clinical study.

15.2 Permission of Access

The Investigator will permit study-related monitoring, audits, IEC review and regulatory inspections, providing direct access to primary patient data (i.e. source data) which supports the data on the CRFs for the study, e.g. general practice charts, hospital notes, appointment books, original laboratory records.

Because this enters into the realm of patient confidentiality, this fact must be included in the Informed Consent Form to be signed by the patient, in line with pertinent data protection legislation.

Any party (e.g. domestic and foreign regulatory authorities, the Sponsor and / or authorised representatives of the Sponsor such as monitors and auditors) with direct access should take all reasonable precautions within the constraints of the applicable regulatory requirements to maintain the confidentiality of patient identities and Sponsor proprietary information.

16 QUALITY CONTROL AND QUALITY ASSURANCE

16.1 Quality Control

16.1.1 Definition

Quality Control is defined as the operational techniques and activities, such as monitoring, undertaken within the quality assurance system to verify that the requirements for quality of the study related activities have been fulfilled.

Quality Control should be applied to each stage of data handling to ensure that all data are reliable and have been processed correctly.

16.1.2 Study Monitoring

Authorized, qualified representatives of the Sponsor will visit investigational sites in regular intervals as defined in the monitoring plan to verify adherence to protocol and

local legal requirements, to perform source data verification and to assist the Investigator in his/her study related activities.

Refer also to section 15.2.

16.2 Quality Assurance

16.2.1 Definition

Quality Assurance is defined as the planned and systematic actions that are established to ensure that the study is performed and the data are generated, documented (recorded) and reported in compliance with Good Clinical Practice (GCP) and the applicable regulatory requirements.

16.2.2 Audit

An audit is a systematic and independent review of study related activities and documents to determine whether the evaluated study related activities were conducted and the data were recorded, analysed and accurately reported according to the protocol, designated Standard Operating Procedure (SOPs), Good Clinical Practice (GCP) and the applicable regulatory requirements.

An independent audit at the study site may take place at any time during or after the study. Refer also to section 15.2.

16.2.3 Inspection

An Inspection is defined as the act by a regulatory authority of conducting an official review of documents, facilities, records and any other resources that are deemed by the authorities to be related to the clinical study and that may be located at the site of the study, or at the Sponsors and / or clinical research organisation facilities or at any other establishments deemed appropriate by the regulatory authorities.

Refer also to section 15.2.

17 ETHICAL AND LEGAL CONSIDERATIONS

17.1 Committees and Boards

17.1.1 Independent Ethics Committee (IEC)

This is an independent body (a review board or a committee, institutional, regional, national or international) constituted of medical / scientific professional and non-medical / non scientific members whose responsibility it is to ensure the protection of the rights, safety and well being of human subjects involved in the study, and to provide public assurance of that protection, by reviewing and providing a favourable opinion on the study protocol, suitability of the Investigator, facilities and the methods and material to be used in obtaining and documenting informed consent from study patients.

The legal status, composition, function, operations and regulatory requirements pertaining to the Independent Ethics Committee may differ among countries, but should allow the Ethics Committee to act in agreement with GCP.

Regarding the conduct of the present study and involvement of IEC refer to section 17.2.

17.1.2 Institutional Review Board (IRB)

This is an independent body constituted of medical scientific and non-scientific members, whose responsibilities is to ensure the protection of the rights, safety and well being of human subjects involved in a study by reviewing approving and providing continued review of the study protocol and amendments and of the methods and material used in obtaining and documenting informed consent of the study patients.

An IRB is not applicable for the present study.

17.1.3 Drug Safety Monitoring Board (DSMB)

A Data Monitoring Board/Committee is a group of independent experts external to a study assessing the progress, safety data and, if needed critical efficacy variable(s) of a clinical study. In order to do so a DSMB may review unblinded study information (on a patient level or treatment group level) during the conduct of the study. Based on its review the DSMB provides the sponsor with recommendations regarding study modification, continuation or termination.

For assessment of the need of a DSMB in a respective study refer to EMEA/CHMP/EWP/5872/03 Corr.

For the present study a DSMB will not be appointed.

17.2 Conduct of Study and Ethical Considerations

This clinical study will be conducted in accordance with the Declaration of Helsinki (see Appendix 1). It will be conducted in compliance with this protocol, Good Clinical Practice (2001/20/EEC, CPMP/ICH/135/95), designated SOPs, and with local laws and regulations relevant to the use of investigational new drugs in the country of conduct.

Before initiating a study, the Investigator should have written and dated approval / favourable opinion from the concerned IEC for the study protocol (and any amendments), written informed consent form, consent form updates, patient recruitment procedures (e.g. advertisements), and any other written information to be provided to patients. Approval will be indicated in writing with reference to the final protocol number and date. Details of the IEC’s constitution including names of its members and their function in the committee (e.g. chairman, specialist, lay-member) should be made available to the Sponsor for inclusion in the Trial Master File.

During the study all documents that are subject to review should be provided to the IEC by the sponsor or the Investigator in line with national provisions.

17.3 Responsibilities

The responsibilities of the Investigator, Monitor and Sponsor of the clinical trial as regards handling of data, storage of data, planning, assessment and quality assurance are regulated by the recommendations on ” ICH Topic E 6 Guideline for Good Clinical Practice” of the ”International Conference on Harmonisation” (ICH) and apply also to this clinical trial.

17.4 General reporting obligation

B. Braun AG shall apply for the authorization of the clinical study at the federal authority and inform local authorities responsible for the Investigators and for the company itself about the planned clinical trial in writing before the beginning of the study.

17.5 Financing and Insurance

The costs necessary to perform the study will be agreed upon with each Investigator and will be documented in a separate financial agreement which will be signed by the Investigator and the Sponsor, prior to the study commencing.

The B. Braun AG has taken out subject insurance with a company of the for all patients taking part in the trial under the policy number .

All investigators shall receive a copy of the insurance certificate and the insurance conditions; the latter must be known to the patients and made available.

17.6 Personal Data and Data Protection

All data obtained in the context of the clinical trial are subject to data protection. The patient’s name in addition to other personal data (excluding date of birth / age and sex) are not to be disclosed by the Investigator.

It must be ensured that CRFs or other documents (e.g. copies of reports on special findings) transmitted to the B. Braun AG contain no names, but only the patients’ study identification (patient number, age and / or a random number).

The storage of data for statistical assessment shall likewise be performed only under the patient’s study identification. Only the Investigator will have the means to identify a patient’s name / other personal details via the study identification.

If it becomes necessary in the course of the study to identify a patient’s name for medical reasons, all individuals involved are subject to an obligation to maintain secrecy.

If personal data are stored and processed, the requirements of pertinent data protection legislation are to be observed.

17.7 Modification of Protocol

The Investigator or the Sponsor should not implement any deviation from, or changes of, the protocol without mutual agreement and in a written form of an amendment to study protocol.. The only exceptions are where necessary to eliminate an immediate hazard to study patients, or when the changes involve only logistical or administrative aspects of the study (e.g. change in monitor(s), change of telephone number(s)). The amendment must be signed and dated by those that signed the first version of the final protocol.

Protocol amendments will be submitted to the concerned IECs and competent authorities in line with pertinent requlatory requirements.

17.8 Investigator's Brochure/Summary of Product Characteristics

The Investigator shall be informed about the preclinical and clinical state of knowledge concerning the Investigational Products.
In the present study this will be done by means of the approved SmPCs of all investigational products. These documents should serve as the basis for the assessment of expectedness of an adverse reaction (see section 11.1.3).

17.9 Completion of Case Report Forms

Any data to be recorded directly in the electronic CRFs (to be considered as source data) will be defined at the start of the study (see section 10.4).

The investigators must ensure the accuracy, completeness, legibility and timeliness of data reported in the CRF and all required reports. Any change or correction to a CRF entry must be explained .

Data reported in the electronic CRF that are derived from source documents should be consistent with the source documents or the discrepancies should be explained.

Within two weeks after completion of each patient, the Investigator should have completed CRF entry available for full inspection by the clinical monitor.

17.10 Archiving

Essential documents are to be retained for the periods required by ICH-GCP, i. e. until at least 2 years after the last approval of a marketing application in an ICH region and until there are no pending or contemplated marketing applications in an ICH region or at least 2 years have elapsed since the formal discontinuation of clinical development of the Investigational Product (CPMP/ICH/135/95), or by national legal requirements, whichever is longer, but not less than 15 years after routine/premature termination of a clinical study.

The final report shall be retained for at least 2 years after the Investigational Products are removed from the last market.The informed consent forms and all the original (raw) data are to be retained by the investigator for at least 15 years.

17.11 Confidentiality

The aim and contents of the study, in addition to its results are to be treated as confidential by all persons involved in the clinical trial.

18 FINAL REPORT AND PUBLICATION POLICY

The Sponsor and Investigator shall agree on the final study report.

It is intended that the results of the study may be published as scientific literature. Results may also be used in submissions to regulatory authorities. The following conditions are to protect commercial confidential materials (patents, etc), not to restrict publication.

All information concerning the investigational products Gelofusine® Balanced, Gelafundin® 4%, Sterofundin® ISO and Sodium chloride 0.9% (such as patent applications, formulae, manufacturing processes, basic scientific data, or formulation information supplied to the Investigator by the Sponsor and not previously published)

is considered confidential and shall remain the sole property of the Sponsor. The Investigator agrees not to use it for other purposes without the Sponsor's written consent.

It is understood by the Investigator that the Sponsor will use the information developed in this clinical study in connection with the development of Gelofusine® Balanced and therefore may be disclosed as required to other Investigators or any appropriate international Regulatory Authorities. In order to allow for the use of information derived from this clinical study, the Investigator understands that he/she has an obligation to provide the Sponsor with complete test results and all data developed during this study.

Prior to submitting the results of this study for publication or presentation, the Investigator will allow the Sponsor 30 days in which to review and comment upon the publication manuscript. The Sponsor agrees that before he publishes any results of this study, he shall provide the Investigators at least 30 days for full review of the publication manuscript. In accordance with generally recognised principles of scientific collaboration, co-authorship with any Sponsor personnel will be discussed and mutually agreed upon before submission of a manuscript to a publisher.

19 REFERENCES

Base E.M., et al., *Efficacy and safety of hydroxyethyl starch 6% 130/0.4 in a balanced electrolyte solution (volulyte) during cardiac surgery.* J Cardiothorac Vasc Anesth, 2011. 25(3): 407-14

Haisch, G., et al., The influence of intravascular volume therapy with a new hydroxyethylstarch preparation (6% HES 130/0.4) on coagulation in patients undergoing major abdominal surgery. Anesth Analg, 2001. 92: p. 565-71

Hogan, JJ, *Colloidal solutions in shock.* JAMA, 1915. 64(9): p. 721-6

Jacobson, S.D., et al., *Gelatin as a substitute for plasma.* Arch Intern Med, 1944. 74: p. 254-7

Kellum, JA, *A balanced view on balanced solutions.* 29th ISICEM Congress, 2009: Abstract

Kieser, M. and Friede, T. (2000): *Blinded sample size reestimation in multiarmed clinical trials*. Drug Information Journal. 34, 455-460

Kieser, M., Friede, T. (2003): *Simple procedures for blind sample size adjustment that do not affect the type I error rate*. Statistics in Medicine 22; 3571-3581

Lundsgaad-Hansen, P. et al., *The therapeutic effects of blood, marodex, physiogel and ringer solution in a standardised model of haemorrhagic shock.* Helvetica Chirurgica cta, 1966. 33: p. 558-90

Morris, C.G. and Low J., *Metabolic acidosis in the critically ill: Part 1. classification and pathophysiology.* Anaesthesia, 2008. 63: p. 294-301

Spahn, D.R., *Efficacy and safety of crystalloids and colloids.* Transfusion Alternatives in Transfusion Medicine, 2000. 2: p. 9-12

Sneyd, R.J., et al., *Safety and tolerability of Volplex® in elective surgery.* Clin Drug Invest, 2004. 24(2): p. 73-79

Wilkes, N.J., et al., *The effects of balanced versus saline-based hetastarch and crystalloid solutions on acid-base and electrolyte status and gastric mucosal perfusion in elderly surgical patients.* Anesth Analg (abbr), 2001. 93(4): p. 811-6

Yunos, N.M., et al., *The biochemical effects of restricting chloride-rich fluids in intensive care (ClinicalTrials.gov NCT 00885404).* Crit Care Med., 2011 June 23

Zander, R., *Fluid management.* Bibliomed – Medizinische Verlagsgesellschaft mgH, 2006. p. 10-11

20 SIGNATURES

The undersigned have read this protocol and agreed to conduct this study in accordance with all stipulations of the protocol and in accordance with the Declaration of Helsinki.

Date	Signature
|__|__|.|__|__|.2012	(Study Manager B. Braun, Statistician – Planning)
|__|__|.|__|__|.2012	(Medical Representative B. Braun)
|__|__|.|__|__|.2012	(Coordinating Investigator /
|__|__|.|__|__|.2012	....
|__|__|.|__|__|.2012	....
