# Supplementary material for: Impact of a new balanced gelatine on electrolytes and pH in the perioperative care
Source: PLoS One. 2019 Apr 29;14(4):e0213057. doi: 10.1371/journal.pone.0213057 (PMC6488052; doi:10.1371/journal.pone.0213057)
Supplement: S1 Table — (DOCX) [file pone.0213057.s003.docx]

S1 Table: Additional markers of coagulation

| Parameter | Balanced | | | unbalanced | | |
| --- | --- | --- | --- | --- | --- | --- |
|  | Baseline | Intra-op | Post-op | Baseline | Intra-op | Post-op |
| FibTEM MCF (mm) | 22.9±10 | -8.1±8.1 | -8.3±8.6 | 23.2±9.1 | -6.4±13.2 | -5.2±14.8 |
| Ap-TEM CT (sec) | 57.6±16.3 | 0.7±17.8 | 11.8±29.3 | 62.2±14.6 | -14.3±18.2 | 0.4±26.7 |
| Ap-Tem MCF (mm) | 67.9±7.9 | -6.9±7.5 | -10.1±14.1 | 67.4±6.5 | -14.3±18.2 | -10.8±11.9 |
| TRAP (U) | 636±520 | -88±272 | -40±792 | 419±384 | 191±622 | 166±462 |
| ASPItest (U) | 510±429 | -175±272.3 | -184±752 | 331±279 | 16±249 | 3.3±236 |
| ADPtest (U) | 608±407 | -228±149 | -261±650 | 320±303 | 104±393 | 153±294^a^ |

ADP test= Thrombocyte aggregation after activation with adenosine diphosphate; Ap-TEM=Aprotinin-TEM (Ex-TEM with aprotinin to inhibit fibrinolysis); ASPI test=Thrombocyte aggregation after activation with arachidonic acid; CT=Clotting time; Fib-TEM=Fibrinogen thrombelastography (Ex-TEM with deactivated platelets); MCF=maximum clot firmness
a p=0.0323 vs. balanced. No other group differences
