# Supplementary material for: Impact of a new balanced gelatine on electrolytes and pH in the perioperative care
Source: PLoS One. 2019 Apr 29;14(4):e0213057. doi: 10.1371/journal.pone.0213057 (PMC6488052; doi:10.1371/journal.pone.0213057)
Supplement: S2 Table — (DOCX) [file pone.0213057.s004.docx]

Table S2: Haemodynamics

| Parameter | Balanced | | | unbalanced | | |
| --- | --- | --- | --- | --- | --- | --- |
|  | Baseline | Intra-op | Post-op | Baseline | Intra-op | Post-op |
| SAP (mmHg) | 134±16 | -9±35 | -27±16 | 134±17 | -9±27 | -20±20 |
| MAP (mmHg) | 93±11 | -12±27 | -21±17 | 95±12 | -10±16 | -17±13 |
| DAP (mmHg) | 72±11 | -13±23 | -19±22 | 75±12 | -11±12 | -16±15 |
| CVP (mmHg) | 11±3 | -4±6 | -6±5 | 10±6 | 1±7 | -4±8 |
| PEEP (cm H_2_O) | 5±2 | 1±2 | 1±0 | 4±2 | 1±2 | -1±7 |
| HR (bpm) | 82±14 | -3±16 | -2±17 | 79±11 | 5±18 | -2±15 |

Bpm=beats per minute;cmH2O=centimeters of water; CVP=central venous pressure; DAP=diastolic arterial pressure; HR=heart rate; MAP=mean arterial pressure; mmHg=millimeters of mercury; PEEP=positive endexpiratory pressure; SAP=systolic arterial pressure
